# Hypoxia-inducible factor (HIF)-3a2 serves as an endothelial cell fate executor during chronic hypoxia

**DOI:** 10.17179/excli2021-4622

**Published:** 2022-02-21

**Authors:** Maciej Jaskiewicz, Adrianna Moszynska, Marcin Serocki, Jaroslaw Króliczewski, Sylwia Bartoszewska, James F. Collawn, Rafal Bartoszewski

**Affiliations:** 1 Department of Biology and Pharmaceutical Botany, Medical University of Gdansk, Gdansk, Poland; 2 Department of Inorganic Chemistry, Medical University of Gdansk, Gdansk, Poland; 3 Department of Cell, Developmental and Integrative Biology, University of Alabama at Birmingham, Birmingham, USA, Birmingham, AL 35233

**Keywords:** hypoxia, human endothelial cells, HIF3A, DDIT4, REDD1

## Abstract

The adaptive response to hypoxia involves the transcriptional induction of three transcription factors called hypoxia inducible factor alpha 1, 2 and 3 (HIF-1α, HIF-2α, and HIF-3α) which dimerize with constitutively expressed beta chains that together form the HIF-1, -2 and -3 transcription factors. During normoxic conditions, the alpha chain is expressed at low levels since its stability is regulated by prolyl-hydroxylation that promotes subsequent ubiquitination and degradation. During hypoxic conditions, however, the prolyl hydroxylases are less active, and the alpha chain accumulates through elevated protein stability and the elevated induction of expression. Two of the three HIFs isoforms present in mammals, HIF-1 and HIF-2, are well characterized and have overlapping functions that promote cell survival, whereas HIF-3's role remains less clear. The HIF-3 response is complicated because the *HIF3A* gene can utilize different promotors and alternate splicing sites that result in a number of different HIF-3α isoforms. Here, using human umbilical vein endothelial cells (HUVECs), we demonstrate that one of the isoforms of HIF-3α, isoform 2 (HIF-3α2) accumulates at a late stage of hypoxia and induces the expression of DNA damage inducible transcript 3 (*DDIT4*), a gene known to promote apoptosis. We also demonstrate that caspase 3/7 activity is elevated, supporting that the role of the HIF-3α2 isoform is to promote apoptosis. Furthermore, we provide evidence that HIF-3α2 is also expressed in seven other primary endothelial cell types, suggesting that this may be a common feature of HIF-3α2 in endothelial cells.

## Abbreviations

*18S* eukaryotic 18S rRNA

BSA bovine serum albumin

DDIT4 *(aka REDD1)* DNA-damage-inducible transcript 4

DMOG dimethyloxalylglycine

ECs endothelial cells

*GLUT1* glucose transporter 1

HCAEC human cardiac artery endothelial cells

HIAEC human iliac endothelial cells

HIF-1 hypoxia-inducible factor 1

HIF-2 hypoxia-inducible factor 2

HIF-3 hypoxia-inducible factor 3

*HIF3A* hypoxia-inducible factor 3 alpha

HIFs hypoxia-inducible factors

HMVEC-D human dermal microvascular endothelial cells

HMVEC-L human lung microvascular endothelial cells

HPAEC human pulmonary artery endothelial cells

HPASMC human pulmonary artery smooth muscle cells

HREs hypoxic-response elements

HUVEC human umbilical vein endothelial cells

miRNA microRNA

mTOR mammalian target of rapamycin

mTORC1 mammalian target of rapamycin complex 1

ODD oxygen-dependent degradation domain

PBS phosphate buffer saline

PHDs prolyl hydroxylases

SDS sodium dodecyl sulphate

SDS-PAGE sodium dodecyl sulphate-polyacryl-amide gel electrophoresis

siRNA small interfering RNA

SMGS smooth muscle growth supplement

TBP TATA-binding protein

UtMVEC human uterine microvascular endothelial cells

VEGFA vascular endothelial growth factor A

## Introduction

Oxygen sensing and providing an adequate cellular response to an unmet O_2_ demand is mediated by the adaptive response to hypoxia. This process is crucial for multicellular organisms and relies on the upregulation of gene networks that restore oxygen homeostasis to facilitate cell survival. The adaptive response is regulated by hypoxia-inducible factors (HIFs) that are the key transcription factors that accumulate in response to low cellular oxygen levels. They bind to hypoxic-response elements (HREs) in the promoters or enhancers of numerous target genes that regulate cell metabolism and survival (Koh and Powis, 2012[19]; Bartoszewski et al., 2019[6]). If the adaptive response proves to be unsuccessful in restoring normal oxygen levels, the cells activate the cellular death pathways (Lenihan and Taylor, 2013[22]; Sendoel and Hengartner, 2014[34]). Understanding the mechanistic details of hypoxia-related cell fate decisions during hypoxia is crucial for the development of efficient therapies for human pathologies including cardiovascular diseases and cancer. The role of HIFs in orchestrating the cell death decision, however, remains poorly understood. Two of the three HIFs isoforms present in mammals, HIF-1 and HIF-2, are well characterized, whereas HIF-3's role remains less clear (Duan, 2016[12]; Ravenna et al., 2016[32]). HIF-3α has been shown to suppress overexpression models of HIF-1 and HIF-2 activity (Hara et al., 2001[14]; Yamashita et al., 2008[39]; Heikkilä et al., 2011[16]), while others assign HIF-3 with partially overlapping functions with HIF-1 and HIF-2 (Heikkilä et al., 2011[16]; Tolonen et al., 2020[38]). Furthermore, the HIF-3 response to hypoxia is complicated by the fact that the *HIF3A* gene can utilize different promotors and alternate splicing sites that result in a large number of short and long isoforms of HIF-3α (Duan, 2016[12]; Ravenna et al., 2016[32]). The only major isoform of HIF-3α expressed in human umbilical vein endothelial cells (HUVECs) is HIF-3α2 (Janaszak-Jasiecka et al., 2016[17]).

In our previous work, we demonstrated that the switch from HIF-1 to HIF-2 constitutes a universal mechanism of human endothelium adaptation to prolonged hypoxia (Bartoszewski et al. 2017[7], 2019[6]). In this model, HIF-1 governs the acute adaptation to hypoxia, whereas HIF-2 activity begins later and creates a transitional switch to govern prolonged hypoxic responses (Bartoszewski et al., 2019[6]; Cabaj et al. 2022[8]). Here, using a panel of 8 different primary human endothelial cell lines, we show that Hif-3α2 protein and HIF-3A mRNA gradually accumulate over time in all of the lines tested. Furthermore, our data suggest that HIF-3α2 accumulation has a proapoptotic function and thus HIF-3α2 is an important component of the cell fate decision during hypoxia.

## Materials and Methods

### Cell culture

Primary human umbilical vein endothelial cells (HUVEC) were purchased from Cellworks (Caltag Medsystems Ltd, UK) and cultured in EGM-2 Bulletkit Medium (Lonza). Primary human cardiac artery endothelial cells (HCAEC), primary human iliac endothelial cells (HIAEC), and primary human pulmonary artery endothelial cells (HPAEC) were purchased from Lonza and cultured in EGM-2 medium. Primary human dermal microvascular endothelial cells (HMVEC-D), primary human lung microvascular endothelial cells (HMVEC-L), primary human uterine microvascular endothelial cells (UtMVEC) were purchased from Lonza and cultured in EGM-2MV medium (Lonza). Primary human pulmonary artery smooth muscle cells (HPASMC) were purchased from Gibco and cultured in Medium 231 supplemented with SMGS (Gibco). Except for HUVECs that were pooled from ten individual donors, all other primary human ECs were obtained from single donors. All primary cell culture experiments were conducted between passages 2 and 6 at a confluence of 70-80 %. 

### Induction of hypoxia

Hypoxia was induced in a CO_2_/O_2_ incubator/chamber specific for hypoxia research (Invivo2 Baker Ruskin). Briefly, cells were cultured in 35 mm or 60 mm dishes (for RNA isolation and protein isolation, respectively) at 0.9 % O_2_ for the time periods specified (PO_2 _was 10-12 mm Hg). Control cells were maintained in normoxia in a CO_2_/O_2_ incubator (Binder).

### Isolation of RNA

Total RNA (containing both mRNA and miRNA) was isolated using miRNeasy kit (Qiagen). RNA concentrations were calculated based on the absorbance at 260 nm. RNA samples were stored at -70 °C until use. 

### Measurement of mRNA quantitative Real-Time PCR (qRT-PCR)

We used TaqMan One-Step RT-PCR Master Mix Reagents (Applied Biosystems) as described previously (Bartoszewska et al., 2017[4], 2019[3]; Janaszak-Jasiecka et al., 2018[18]) using the manufacturer's protocol (retrotranscription: 15 min, 48 ºC). The relative expressions were calculated using the 2^-ΔΔCt^ method (Livak and Schmittgen, 2001[25]) with the TATA-binding protein (*TBP*), ribosomal protein lateral stalk subunit P0 (*RPLP0*) and *18S rRNA *genes as reference genes for the mRNA. TaqMan probes ids used were: *18S* (Hs99999901_s1); TBP (Hs00427620_m1); *RPLP0* (Hs00420895_gH).

### Western blots

Cells were lysed in SDS lysis buffer (4 % SDS, 20 % glycerol, 125 mM Tris-HCl pH = 6.8) supplemented with protease inhibitors (cOmplete^TM^ Mini (Roche)). The insoluble material was removed by centrifugation at 15,000 g for 15 min. Protein concentrations were determined by BioRad™ DC-Protein Assay using bovine serum albumin (BSA) as standard. Following the normalization of protein concentrations, the lysates were mixed with an equal volume of 2X Laemmli sample buffer and incubated for 5 min at 95 °C before separation by SDS-PAGE on Criterion TGX stain-free 4-15 % gradient gels (BioRad). Following SDS-PAGE, the proteins were transferred to polyvinylidene fluoride membranes (30V overnight at 4 °C). The membranes were then blocked with BSA (Sigma-Aldrich) dissolved in PBS/Tween-20 (3 % BSA, 0.5 % Tween-20 for 1-2 hours), followed by immunoblotting with the primary antibody: rabbit anti-HIF-3α (Sigma-Aldrich, AV39936 (1:800)); rabbit anti-β-Actin (1:1000, ab1801; Abcam). The rabbit anti-HIF-3α antibody only recognizes the α1, α2 and α3 isoforms of HIF-3α. After the washing steps, the membranes were incubated with goat anti-rabbit IgG (H+L chains) or HRP-conjugated secondary antibodies (BioRad) and detected using SuperSignal West Pico ECL (Thermo Fisher Scientific). Densitometry was performed using Image Lab software v. 4.1 (BioRad).

### siRNA transfections

siRNA against *HIF3A* (Ambion assay id s34653) was purchased from Ambion. siRNAs target 3 *HIF3alpha* isoforms - *HIF3A variant 2* (NM_0224624), *HIF3A variant 1* (NM_15794) and *HIF3A variant 3* (NM_15279). Based on NGS in HUVECs and conformation with real-time PCR, we previously reported that only *HIF3A2* and *HIF3A3* are expressed in HUVECs, with *HIFA2* being the major form expressed (Ravenna et al., 2014[31]). HUVEC cells were transfected using the Lipofectamine RNAiMax (Invitrogen, 13778030) according to the manufacturer's protocol. The siRNAs were used at final concentrations of 40 nM. The transfected cells were cultured for 2 days prior to further analysis. The degree of *HIF3A* knockdowns was determined by qPCR. Silencer Select Negative Control 1 (assay id MC22484) was used as a control.

### Monitoring prolyl hydroxylase activity

To monitor prolyl hydroxylase-dependent degradation of Hif-3α, we used reporter vector based on pEZX-FR02 cloning vector (GeneCopoeia) that contained *HIF3A *(NM_152794) gene region comprising ODD domain (oxygen-dependent degradation domain, amino acids 450-576) fused with firefly luciferase (without the ATG start codon) in-frame downstream of the *HIF3A* and renilla luciferase (expressed from different promoter) as a transfection control (Stiehl et al., 2006[37]; Smirnova et al., 2018[36]; Günter et al., 2020[13]). As a control unmodified pEZX-FR02 vector was used. Briefly, 24 hours after transfection, the cells were seeded onto 96-well luminescence assay white plates with clear bottoms (Corning Inc., 3903) (Stiehl et al., 2006[37]; Smirnova et al., 2018[36]; Günter et al., 2020[13]). The next day the cells were exposed to hypoxia for indicated time points or to Dimethyloxalylglycine; N-(methoxyoxoacetyl)-glycine (DMOG, Sigma, D3695), and luciferase activity measured with Dual-Luciferase Reporter Assay System (Promega) in accordance with the manufacturer's instructions (GloMax-Multi + Detection System, Promega). Each time point was assayed in 6 technical replicates, experiments were repeated in 2 biological replicates.

### Monitoring caspase 3 and caspase 7 activity

Caspase 7 is considered to be redundant with caspase 3 because these enzymes share an optimal peptide recognition sequence and have several endogenous protein substrates in common (Lamkanfi and Kanneganti, 2010[21]). Furthermore, both of these enzymes are activated by the initiators caspase 8 and caspase 9 (Lamkanfi and Kanneganti, 2010[21]). While our main goal was to assess caspase 3 activity, the commercially available assays do not distinguish between these two cysteine proteases. Therefore, we used the caspase-Glo 3/7 assay (Promega) to determine relative caspase activity. Briefly, the day after transfection with the specified siRNA, cells were seeded onto 96-well luminescence assay white plates with clear bottoms (Corning Inc., 3903). The next day cells were exposed to hypoxia for indicated time points. Following treatment, cells were washed with PBS and the Caspase-Glo 3/7 assay (Promega) was performed in accordance with the manufacturer's instructions using GloMax-Multi + Detection System (Promega). Each time point was assayed in 6 technical replicates, experiments were repeated in 3 biological replicates. The results from biological replicates were normalized to the values obtained from the respective non-transfected control cells.

### Statistical analysis

Results were expressed as a mean ± standard deviation. Statistical significance was determined using the Student's t-test with P < 0.05 considered significant. The correlation was accessed via the Spearman correlation method. 

## Results

### The HIF-3α2 accumulates in human endothelial cells exposed to prolonged hypoxia

**Figure 1:**
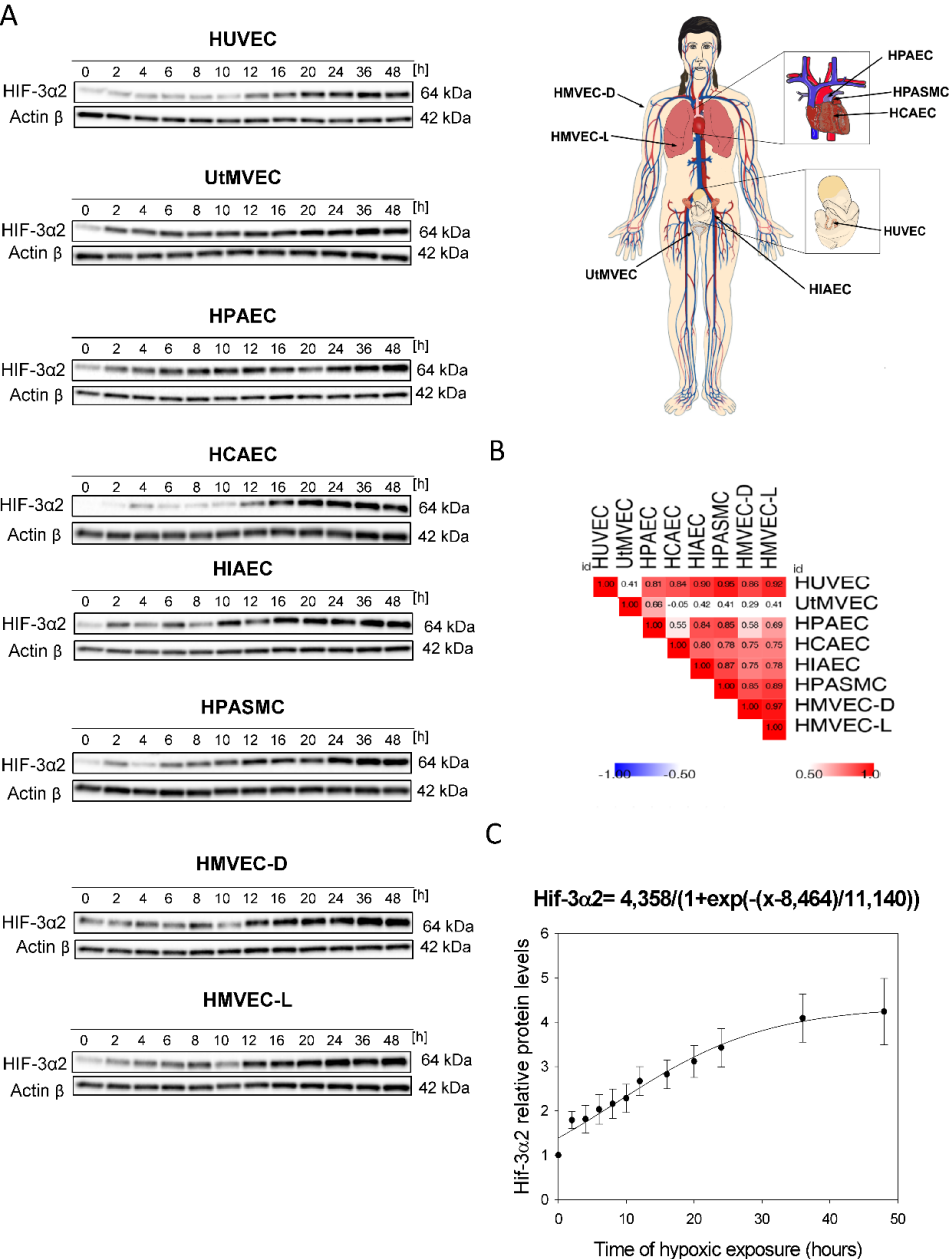
Prolonged hypoxia results in the accumulation of HIF-3α2 in human HUVECs and other endothelial cell types. HUVEC, UtMVEC, HPAEC, HCAEC, HIAEC, HPASMC, HMVEC-D and HMVEC-L, cells were exposed to hypoxia for the time periods specified and total RNA and protein lysates were collected. (A) The changes in HIF-3α2 protein levels were evaluated by western blot normalized to β-actin and total protein levels and related to the normoxic control. In all of the endothelial cell types, the HIF-3α2 isoform is expressed. The HIF-3α2 protein level analysis was performed in parallel with HIF-1α and HIF-2α analyses that were presented in Bartoszewski et al. (2019). The raw data are presented in Supplementary data (PDF). (B) Spearman's correlation of the hypoxia induced HIF-3α2 protein levels change in different human ECs. The mathematic representation of HIF-3α2 changes in human ECs exposed to hypoxia. (C) The changes in HIF-3α levels (from all ECs tested) during hypoxia time course were analyzed using the sigmoid 3-parameter function (using 200 iterations, P < 0.005). The error bars represent SE.

**Figure 2:**
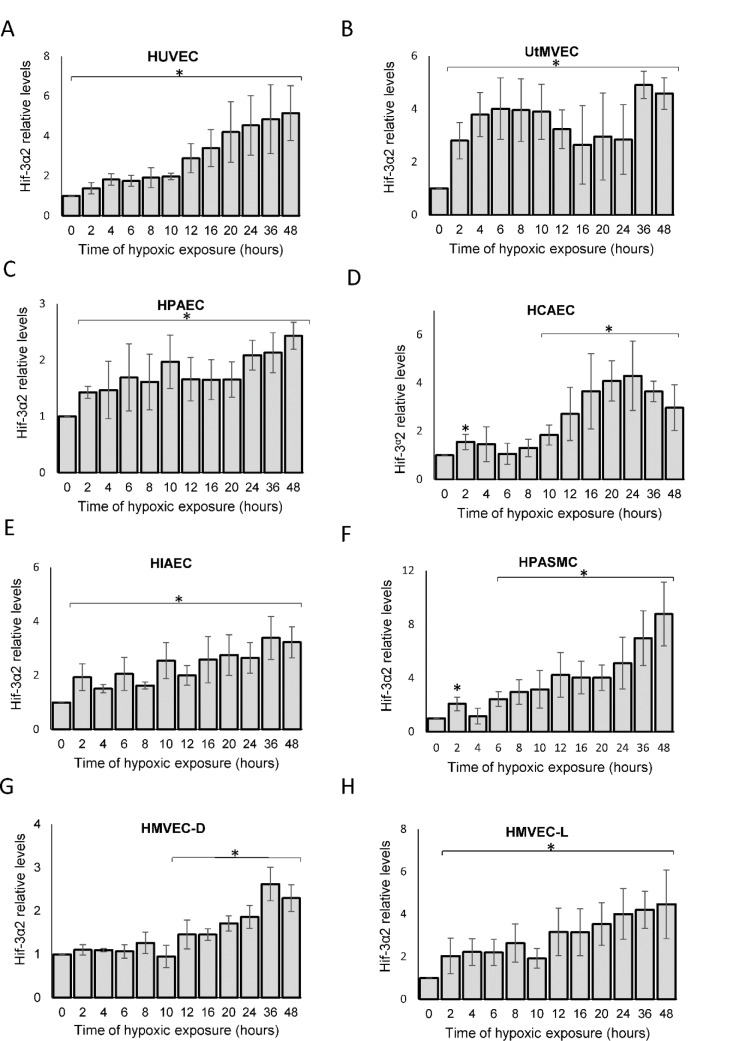
The densitometry analyses of the hypoxic changes in HIF-3α2 levels in human endothelium during hypoxia*. *The changes in HIF-3α2 protein levels were evaluated by western blot normalized to β-actin and total protein levels and expressed as a fold change over the normoxic control. Data represent the mean ± SD of three independent experiments. * P < 0.05 was considered significant. The raw data are presented in Supplementary data (XLSX).

In a previous study, we reported that the predominant HIF-3α´2 isoform (NM_022462.4, 64 kDa) accumulates in HUVECs exposed to chronic hypoxia (Janaszak-Jasiecka et al., 2016[17]). Our goal here was to determine whether HIF-3α2 accumulation also occurs in other ECs and to understand the kinetics of hypoxia-induced changes in HIF-3α levels. To accomplish this, as shown in Figures 1(Fig. 1) and 2(Fig. 2), we performed parallel time-course studies during hypoxia in eight human primary endothelial cell lines of different vascular beds.

This analysis indicated that HIF-3α2 gradually accumulates during acute hypoxia in HUVECs and a similar pattern occurred in all the tested ECs. The 64 kDa protein is consistent with the MW of the HIF-3α2 isoform, whereas the HIF3-α1 and HIF3-α3 isoforms have a molecular weight of about 72 kDa, and this band was not detected. Interestingly, the hypoxic HIF-3α2 accumulation was delayed (after about 12 hours) in HUVECs, HCAEC, HPASMC and HMVEC-D, whereas in the rest of the studied ECs, there was a significant increase in HIF-3α2 protein levels even after 2 hours of exposure to hypoxia. Nevertheless, HIF-3α2 hypoxic expression profiles were well correlated between almost all of the ECs (Figure 1B(Fig. 1)). The exception was for the UtMVEC, where HIF-3α2 accumulated in a similar manner but in a significantly higher amount than in the other ECs. Notably, in terms of HIF-3α2 expression changes, there did not appear to be any general differences nor trends between vascular and microvascular ECs. Taken together, this analysis confirmed that in all human ECs tested, hypoxia results in accumulation of HIF-3α2 after prolonged hypoxia. 

Based on these results, we propose a general model of the HIF-3α2 accumulation in human ECs exposed to hypoxia, in which we calculated the maximum protein expression levels for HIF-3α using the sigmoid 3 parameter function (Figure 1C(Fig. 1)). The beginning of the accumulation of the HIF-3α2 in ECs is observed at about 8 hours (P=0.0001) of hypoxia exposure and reaches a maximum at 20-24 hours (Figure 1C(Fig. 1)). The quantitation of the HIF-3α2 expression levels during the time course is shown in Figure 2(Fig. 2).

### The hypoxic HIF-3α2 accumulation results from HIF3A mRNA induction and impaired prolyl hydroxylase activity 

**Figure 3:**
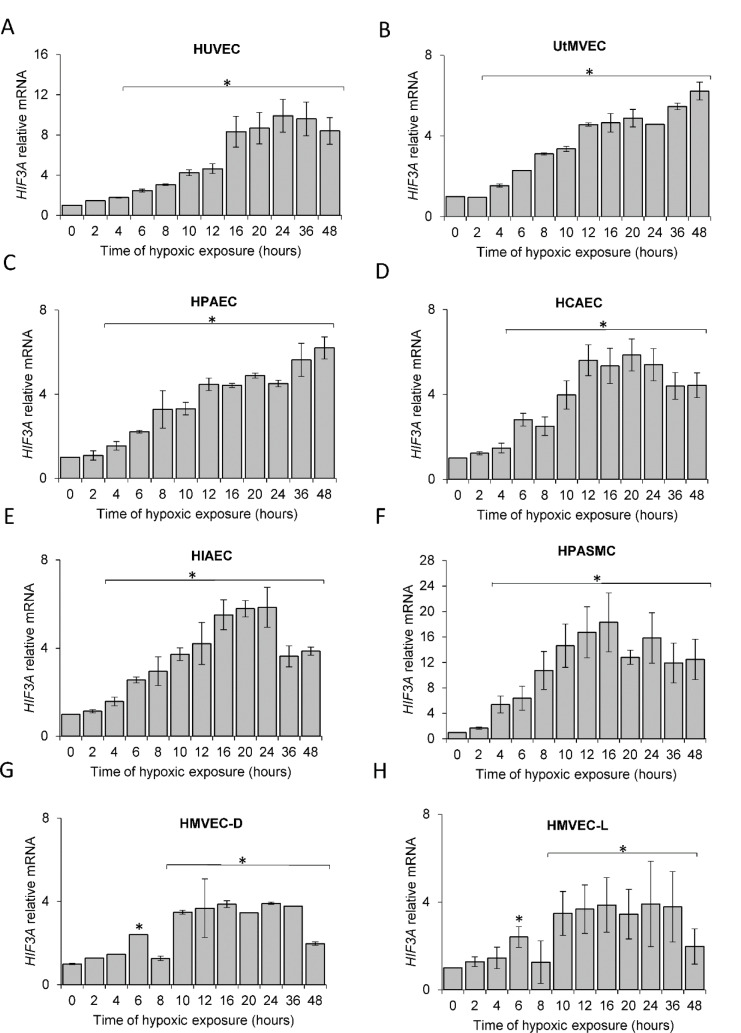
Hypoxia induces *HIF3A* mRNA levels in human ECs. Cells were exposed to hypoxia for the time periods specified and total RNA lysates were collected. *HIF3A* mRNA levels were quantified by qRT-PCR and normalized to *TBP *and *RPLP0* RNA levels and expressed as a fold change over normoxic samples. Data represent the mean ± SD of three independent experiments. * P < 0.05 was considered significant. The raw data are presented in Supplementary data (XLSX).

**Figure 4:**
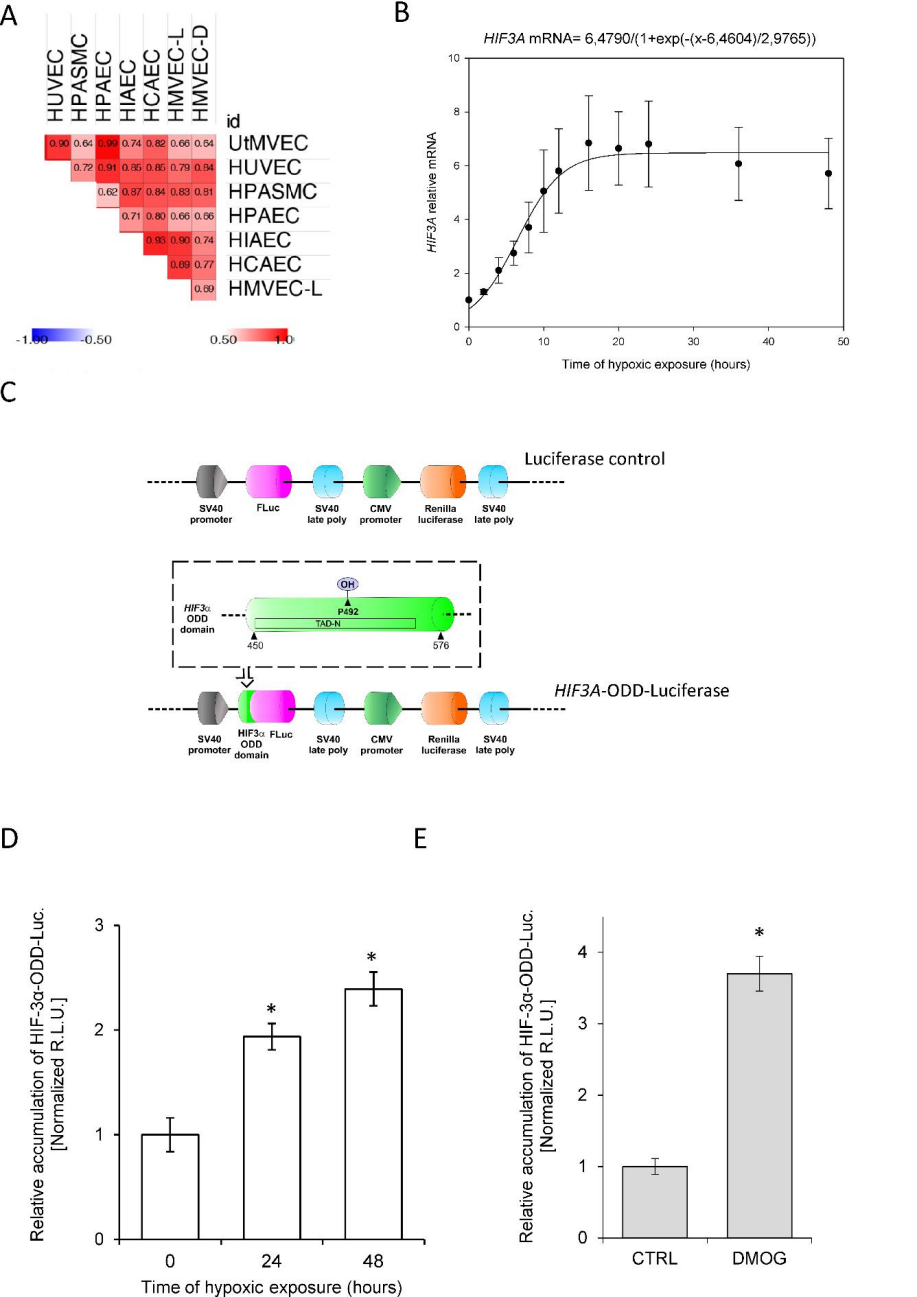
Hypoxic accumulation of HIF-3α results from both increased expression and prolyl hydroxylase inactivation in HUVECs. (A) The Spearman's correlation of the hypoxia induced-*HIF3A *mRNA level changes in different human ECs. (B) The mathematic representation of *HIF3A* mRNA levels during hypoxia in human ECs. The changes in mRNA levels obtained from all ECs tested during the hypoxia time course were analyzed The changes in *HIF3A* levels in HUVECs during hypoxia time course were analyzed using the sigmoid 3 parameter function (using 200 iterations, P < 0.005). The error bars represent SE. (C) Schematic presentation of firefly luciferase (FLuc) and renilla luciferase reporter cloning vector pEZX-FR02 (GeneCopoeia) containing the *HIF3A* human (NM_152794) gene region comprising ODD (amino acids 450-576, including the Proline 492 (a PHD substrate) was fused with firefly luciferase (without the ATG start codon) in-frame downstream of the *HIF3A*. (D) Hypoxia-related stabilization of the HIF-3α ODD reporter. HUVECs following the transfection with the HIF-3α-ODD reporter vector were exposed to hypoxia for indicated time points and luciferase activity measured with Dual-Luciferase Reporter Assay System. The data were normalized to the control vector (without HIF-3α-ODD) and expressed as a fold change over the normoxic signal. R.L.U. - relative light units, Data represent the mean ± SD of three independent experiments (6 replicates each). * P < 0.05 was considered significant. (E) PHD inhibition-related stabilization of HIF-3α ODD reporter. HUVECs following the transfection with the HIF-3α-ODD reporter vector were treated with 1 mM DMOG for 16 hours and luciferase activity was measured with Dual-Luciferase Reporter Assay System. The data were normalized to control vector (without HIF-3α-ODD) and expressed as a fold change over the normoxic signal. R.L.U. - relative light units, Data represent the mean ± SD of two independent experiments (4 replicates each). * P < 0.05 was considered significant. The raw data are presented in Supplementary data (XLSX).

Although previous studies have reported that *HIF3A* mRNA levels are increased during hypoxia in human ECs (Augstein et al., 2011[2]), our goal was to understand the kinetics of these hypoxia-induced changes. To accomplish this, we performed parallel time-course studies during hypoxia in the same set of human primary ECs and found that *HIF3A *mRNA is gradually induced at 6 hours in all ECs (Figure 3(Fig. 3)), reached maximum levels at 10 to 16 hours, and remained elevated for 48 hours. Similar to the HIF-3α2 protein results, the *HIF3A *mRNA profiles were significantly correlated in all ECs (Figure 4A(Fig. 4)), however, the correlation was less pronounced than that of the protein. In this case, the RNA profiles could be for the *HIF3A1*, *HIF3A2* or *HIF3A3* isotypes given that the primers cannot distinguish between these three isoforms. Hence, we have labeled these as *HIF-3A*.

Based on these results, we propose a general model of the *HIF3A* hypoxic profile in human ECs, using the sigmoid 3 parameter function (Figure 4B(Fig. 4)), and noted that *HIF3A* mRNA accumulation begins after about 7 hours of exposure to hypoxia (P=0.0001) and reaches a maximum level at about 15 hours. Notably, this model is in good agreement with the one obtained for the HIF-3α2 protein, showing that both mRNA and protein are accumulated during hypoxia in a correlated manner (**a** coefficient for the protein 4.358 P=0.0001, **a** coefficient for the mRNA 6,479 P=0.0001), with protein reaching the maximum levels earlier than the mRNA. This observation is consistent with the hypothesis that *HIF3A* mRNAs induction contributes to the HIF-3α2 protein accumulation in hypoxia-exposed ECs.

Besides mRNA induction, hypoxic impairment of prolyl hydroxylase (PHDs) activity is also considered as a canonical mechanism responsible for HIF subunit accumulation during low oxygen conditions (Semenza, 2000[33]; Metzen and Ratcliffe, 2004[26]). To follow this process in the HIF-3α context, we next monitored the PHDs activity against HIF-3α using a reporter vector containing HIF-3α oxygen degradation domain (ODD) fused with luciferase (Figure 4C(Fig. 4), *HIF3A*-*ODD-Luciferase*) in HUVECs cultured in normoxia and during hypoxia (Stiehl et al., 2006[37]; Smirnova et al., 2018[36]; Günter et al., 2020[13]). We observed, that the HIF-3α-ODD-luciferase accumulates in hypoxic conditions (Figure 4D(Fig. 4)), as well as when PHD activity was impaired with the specific inhibitor, DMOG (Yuan et al., 2014[41]) (Figure 4E(Fig. 4)). Since the maximum level of HIF-3α-ODD accumulation during hypoxia is about half of that observed for the protein HIF-3α, our data would suggest that for the increased HIF-3α protein expression in ECs, both the induction of *HIF3A *mRNA and inhibition of PHD-dependent degradation are equally important.

### The HIF-3α2 effects on the HUVEC apoptotic signaling during hypoxia

**Figure 5:**
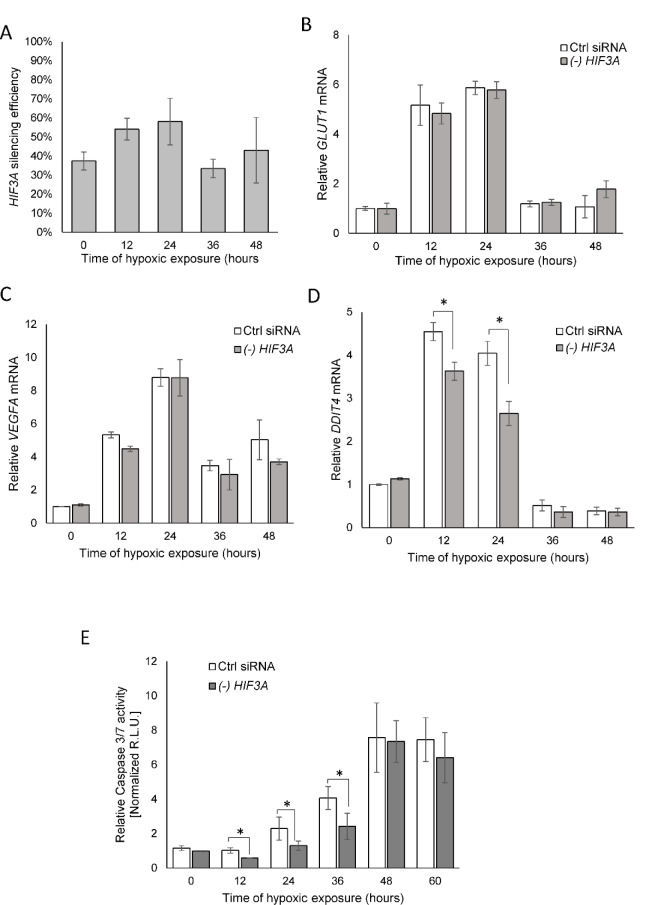
HIF-3α contributes to hypoxia-related elevated caspase 3 levels in HUVECs. (A) The silencing efficiency of *HIF3A *in hypoxia exposed HUVECs. Cells were transfected with siRNA against the *HIF3A* or control siRNA and exposed to hypoxia for the time periods specified and total RNA lysates were collected. *HIF3A* mRNA levels were quantified by qRT-PCR and normalized to *TBP *and *18S* rRNA levels and expressed as a percentage of control siRNA transfected samples. Data represent the mean ± SD of two independent experiments (3 replicates each). * P < 0.05 was considered significant. (B) *HIF3A* knockdown does not affect hypoxia-induced expression of *GLUT1* and (C) *VEGFA*, but does reduce (D) *DDIT3* mRNA levels in hypoxia exposed HUVECs. (E) The caspase 3/7 activity was monitored by luminescence and expressed in Relative Light Units (R.L.U.). HUVECs with *HIF3A* knockdown or transfected with control siRNA for each time point of exposure to hypoxia were seeded in six technical replicates, and the experiments were repeated three times. Error bars represent standard deviations, *P < 0.05 was considered significant. The raw data are presented in Supplementary data (XLSX).

Although the hypoxic accumulation of HIF-3α was previously reported, the role of HIF-3α's response to hypoxia is still controversial. It has been reported that some HIF-3α variants, due to the absence of the transactivation domains, counteract the transcriptional activity of HIF-1α and HIF-2α (Hara et al., 2001[14]; Yamashita et al., 2008[39]; Heikkilä et al., 2011[16]). Given that, we followed the consequences of a *HIF3A* knockdown on the well-established HIF-1 and HIF-2 transcriptional targets of *VEGFA* and *GLUT1 *mRNA levels (Liu et al., 1995[24]; Chen et al., 2001[10]; Kurihara et al., 2014[20]) in hypoxia-exposed HUVECs. In agreement with the previous studies (Tolonen et al., 2020[38]), *HIF3A* silencing during hypoxia was less efficient than in normoxic conditions (about 50 % compared to 70 %, respectively, Figure 5A(Fig. 5)). As shown in Figures 5B and C(Fig. 5), the *HIF3A* mRNA knockdown had no effect on *VEGFA* or* GLUT1* expression during the hypoxia time course. Notably, however, *HIF3A* silencing in HUVECs exposed to hypoxia from 12 to 24 hours resulted in a reduction of the expression of the DNA damage inducible transcript 4 (*DDIT4, *aka *REDD1*) that has been previously reported to be a HIF-3 transcriptional target (Zhang et al., 2014[42]; Janaszak-Jasiecka et al., 2016[17]) (Figure 5D(Fig. 5)). Given that DDIT4 function inhibits the mammalian target of rapamycin (mTOR) during stress, and DDIT4 expression promotes tumor cell apoptosis (Yang et al., 2018[40]), we next tested if *HIF3A* knockdown resulted in increased HUVECs survival as monitored by decreased caspase activity during hypoxia because of reduced *DDIT4* expression. As shown in Figure 5E(Fig. 5), the reduced *HIF3A *expression during hypoxia significantly reduced caspase 3/7 activity up to 36 hours and suggested that HIF-3α2 accumulation during hypoxia would stimulate elevated apoptosis through elevated levels of caspase 3/7. Starting from 48 hours, we observed the maximal levels of caspase activity in both control and *HIF3A *silenced cells, which corresponded well with the reduction of HIF transcriptional target expression (Figure 5(Fig. 5)). 

Taken together, our results show that accumulation of HIF-3α2 in HUVECs serves as a continuously accumulating apoptotic signal and thus provides a molecular timer for cell fate decisions.

See also the Supplementary data .

## Discussion

In order to take advantage of regulating endothelial cell fate during hypoxia as a possible therapeutic intervention, it is important to understand the molecular pathways governing this process and especially the role of HIF-1 and HIF-2 that have been predominately assigned to cellular adaptation and survival during hypoxia (Ratcliffe, 2007[30]; Albadari et al., 2019[1]). Less is known about the role of HIF-3 due in part to the presence of multiple HIF-3A variants (Duan, 2016[12]; Ravenna et al., 2016[32]). Despite the fact that some short HIF-3A isoforms have been proposed to be dominant-negative regulators of HIF-1 and 2 activity (Hara et al., 2001[14]; Yamashita et al., 2008[39]; Heikkilä et al., 2011[16]), the full-length HIF-3α protein functions were reported to activate a unique transcriptional program in response to hypoxia (Heikkilä et al., 2011[16]; Tolonen et al., 2020[38]). Our understanding of the HIF-3's role in the adaptive response to hypoxia in human endothelial cells, however, remains very limited. In our previous studies, we demonstrated that the transition from HIF-1α to HIF-2α constitutes a universal mechanism of cellular adaptation to hypoxia in human ECs (Bartoszewska et al., 2015[5], 2019[3]). Using a similar approach, we show here that HIF-3α2 protein, which is the dominant one in HUVECs (Janaszak-Jasiecka et al., 2016[17]), accumulates gradually during the time of hypoxic exposure in all of the ECs. These data are in agreement with the previous reports showing that most of the protein HIF-3α variants accumulate during hypoxia (Augstein et al., 2011[2]). Furthermore, HIF-3α2 has been shown to have transcriptional activity and contribute to the hypoxic increase in erythropoietin (*EPO*) expression in Hep3B and the SK-N-AS neuroblastoma cell lines that are capable of endogenous EPO production (Tolonen et al., 2020[38]). Although our previous studies indicated that HIF-1α expression during hypoxia differed between vascular and microvascular ECs (Bartoszewski et al., 2019[6]), this analysis of HIF-3α changes during hypoxia, despite some small differences between individual cell lines, appeared to be similar in both vascular and microvascular ECs. 

To understand the mechanism responsible for the HIF-3α2 accumulation, we performed mRNA time-course studies during hypoxia in the same set of endothelial cell lines. We observed that the hypoxic induction of *HIF3A* mRNA in all tested ECs had kinetics correlated well with the HIF-3α2 protein profiles. Our data are in good agreement with previous studies demonstrating elevated *HIF3A* expression elevation during hypoxia was the result of both transcriptomic induction of *HIF3A* expression by both HIF-1 and HIF-2 (Augstein et al., 2011[2]). HIF-3 elevation could also be due to reduced levels of miRNA-429 which targets *HIF3A* mRNA (Janaszak-Jasiecka et al., 2016[17]). Since most HIF-3α variants contain a single proline that can be hydroxylated in their ODD domain (Duan, 2016[12]; Ravenna et al., 2016[32]), these proteins could also potentially accumulate during hypoxia due to PHD inactivation. In that regard, previous reports in other cell types, however, have questioned whether the single proline could effectively direct some HIF-3α isoforms for proteasomal degradation (Pasanen et al., 2010[29]; Ravenna et al., 2014[31]). These observations are in contrast to the other reports showing accumulation of HIF-3α isoforms with PHDs inhibitor treatments (Heidbreder et al., 2003[15]; Li et al., 2006[23]; Pasanen et al., 2010[29]; Ravenna et al., 2016[32]). Here using HUVECs with the dedicated HIF-3α-ODD reporter, we show that inhibition of PHD-dependent HIF-α degradation as well as hypoxia can significantly contribute to HIF-3α accumulation. Our results supported this hypothesis, although this oxygen-dependent HIF-3α stabilization is probably not sufficient by itself to explain the magnitude of protein induction. Hence, both mRNA induction and impairment of PHD activity seem quite plausible as a mechanism responsible for HIF-3α accumulation during hypoxia. The importance of each of these stages will obviously require further study. Furthermore, previous studies usually focused on a single cell line, and often indicated cell type specific kinetics of HIF-3α induction at both mRNA and protein levels (Duan, 2016[12]; Ravenna et al., 2016[32]). 

Since the functional role of HIF-3 in response to hypoxia is obviously variant- and model-dependent (Duan, 2016[12]; Ravenna et al., 2016[32]), here we focused on HIF-3α2's role on HUVECs, which previously indicated that this isoform was the dominant one (Janaszak-Jasiecka et al., 2016[17]). Since other reports indicated that some other long HIF-α isoforms could negatively affect HIF-1 and HIF-2 transcriptional activity, we tested this possibility in hypoxia exposed ECs with and without *HIF3A* knockdown, followed by determining mRNA levels of the well-established HIF-1 and HIF-2 transcriptional targets, *VEGFA* and *GLUT1 *(Liu et al., 1995[24]; Chen et al., 2001[10]; Kurihara et al., 2014[20]). Importantly, we did not observe any significant increase in their hypoxic expression when *HIF3A *was silenced, suggesting that in human ECs, HIF-3α does not limit HIF-1 or HIF-2 activity (Bartoszewski et al. 2019[6]). Interestingly, although unaffected by *HIF3A *knockdown in HUVECs, the *GLUT1 *was proposed as a weak transcriptional target of HIF-3α9 in Hep3B (Heikkilä et al., 2011[16]) cells*.* Numerous HIF-3α effects, however, have been shown to be isoform and cell type specific. The *DDIT4* transcript that was originally reported as a long HIF-3α isoforms target in zebrafish (*Danio rerio*) embryos (Zhang et al., 2014[42]) was also proposed by us as a HIF-3α dependent transcript in HUVECs (Janaszak-Jasiecka et al., 2016[17]). In these studies, it was significantly reduced upon HIF-3α knockdown in hypoxia exposed HUVECs. Furthermore, DDIT4 was also reported to be increased in U2OS osteosarcoma and HEK293 cells following HIF-3α9 stabilization (Ravenna et al., 2016[32]). DDIT4 is reported to be a potent inhibitor of mTOR, and an increase in DDIT4 can induce apoptosis (Shoshani et al., 2002[35]; Cam et al., 2010[9]; Noseda et al., 2013[27]; Ota et al., 2014[28]; Chen et al., 2016[11]; Yang et al., 2018[40]). Based on that, we tested if HIF-3α2 altered the caspase 3 levels of ECs during hypoxia. Notably, the *HIF3A* knockdown resulted in significantly lower caspase activation in hypoxia exposed HUVECs up to 36 hours. Hence, it is very plausible that HIF-3α2 accumulation in hypoxia exposed ECs leads to DDIT4-related mTOR inhibition and activation of apoptosis. However, the exact molecular mechanism of the HIF-3α2-related induction of hypoxic cell death will require further study.

Taken together, our study demonstrates that the accumulation HIF-3α2 is a part of human endothelial response to prolonged hypoxia and results from both increased *HIF3A* mRNA and impaired PHD degradation of HIF-3α. Furthermore, we show that the gradual accumulation of HIF-3α in ECs eventually leads to increased levels of caspase 3 when the adaptive response to hypoxia fails. Hence, HIF-3α2 serves as a molecular timer of cell fate decisions during prolonged hypoxia in HUVECs and perhaps other endothelial cell types as well.

## Declaration

### Author contributions

Conceptualization, Rafal Bartoszewski; Formal analysis, Rafal Bartoszewski; Funding acquisition, Rafal Bartoszewski; Investigation, Rafal Bartoszewski, Maciej Jaśkiewicz, Adrianna Moszyńska, Marcin Serocki, Jarosław Króliczewski, Sylwia Bartoszewska. Methodology, Rafal Bartoszewski and Jarosław Króliczewski; Project administration, Rafal Bartoszewski; Writing - original draft, Rafal Bartoszewski, James F. Collawn; Writing - review & editing, Rafal Bartoszewski, Sylwia Bartoszewska, Jarosław Króliczewski, Maciej Jaśkiewicz and James F. Collawn.

### Funding

This research was funded by the National Science Center "SONATA BIS" Program under contract UMO-2015/18/E/NZ3/00687(R.B.). J.F.C. was funded by an NIH P30 DK072482 and a Research Development Program Grant from the Cystic Fibrosis Foundation.

### Conflicts of interest

The authors declare no conflict of interest.

## Supplementary Material

Supplementary data PDF

Supplementary data XLSX
