# Supplementary material for: Hypoxia-inducible factor (HIF)-3a2 serves as an endothelial cell fate executor during chronic hypoxia
Source: EXCLI J. 2022 Feb 21;21:454–69. doi: 10.17179/excli2021-4622 (PMC8983852; doi:10.17179/excli2021-4622)

EXCLI Journal 2022;21:454-469 – ISSN 1611-2156
Received: December 20, 2021, accepted: February 08, 2022, published: February 21, 2022

Original article:

HYPOXIA-INDUCIBLE FACTOR (HIF)-3α2 SERVES AS AN ENDOTHELIAL CELL FATE EXECUTOR DURING CHRONIC HYPOXIA

Maciej Jaśkiewicz[1], Adrianna Moszyńska[1], Marcin Serocki[1], Jarosław Króliczewski[1], Sylwia Bartoszewska[2], James F. Collawn[3], Rafal Bartoszewski[1*]

[1] Department of Biology and Pharmaceutical Botany, Medical University of Gdansk, Gdansk, Poland
[2] Department of Inorganic Chemistry, Medical University of Gdansk, Gdansk, Poland
[3] Department of Cell, Developmental and Integrative Biology, University of Alabama at Birmingham, Birmingham, USA, Birmingham, AL 35233

* **Corresponding author:** Rafal Bartoszewski, Department of Biology and Pharmaceutical Botany, Medical University of Gdansk, al. Gen. J. Hallera 107, 80-416 Gdansk, Poland; Tel: 48 58 349 32 14; Fax: 48 58 349 32 11; E-mail: rafalbar@gumed.edu.pl

https://dx.doi.org/10.17179/excli2021-4622

This is an Open Access article distributed under the terms of the Creative Commons Attribution License (http://creativecommons.org/licenses/by/4.0/).

Raw data for Figure 1

HUVEC

Replicate 1

HIF-3α2

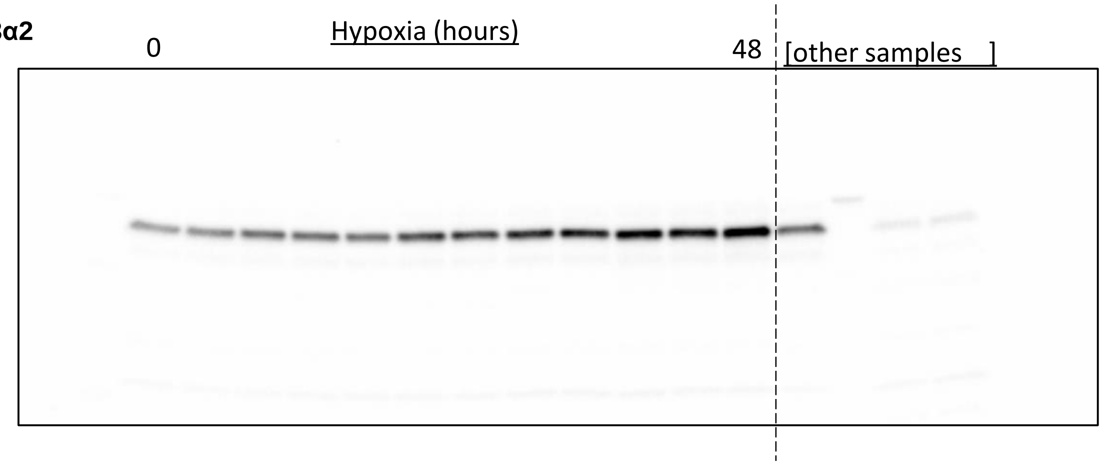

Actin β

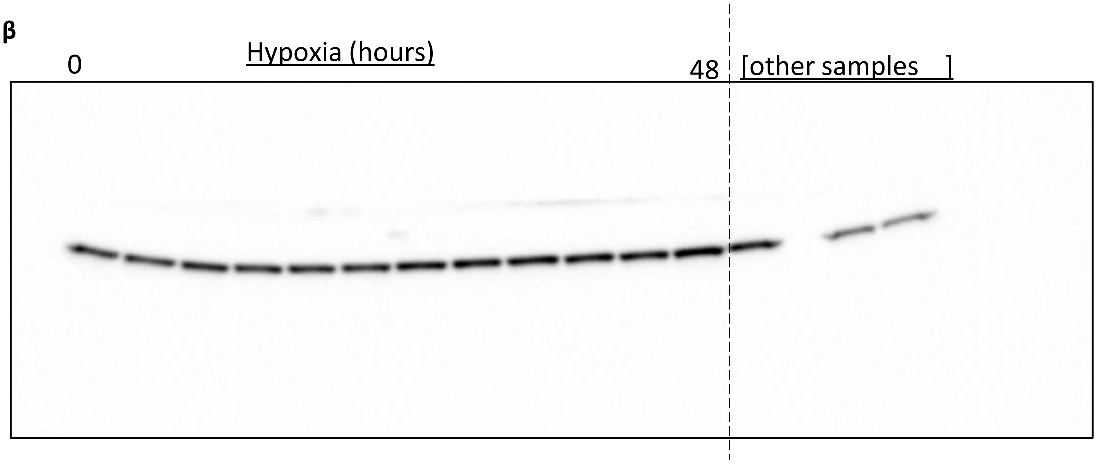

Total Protein

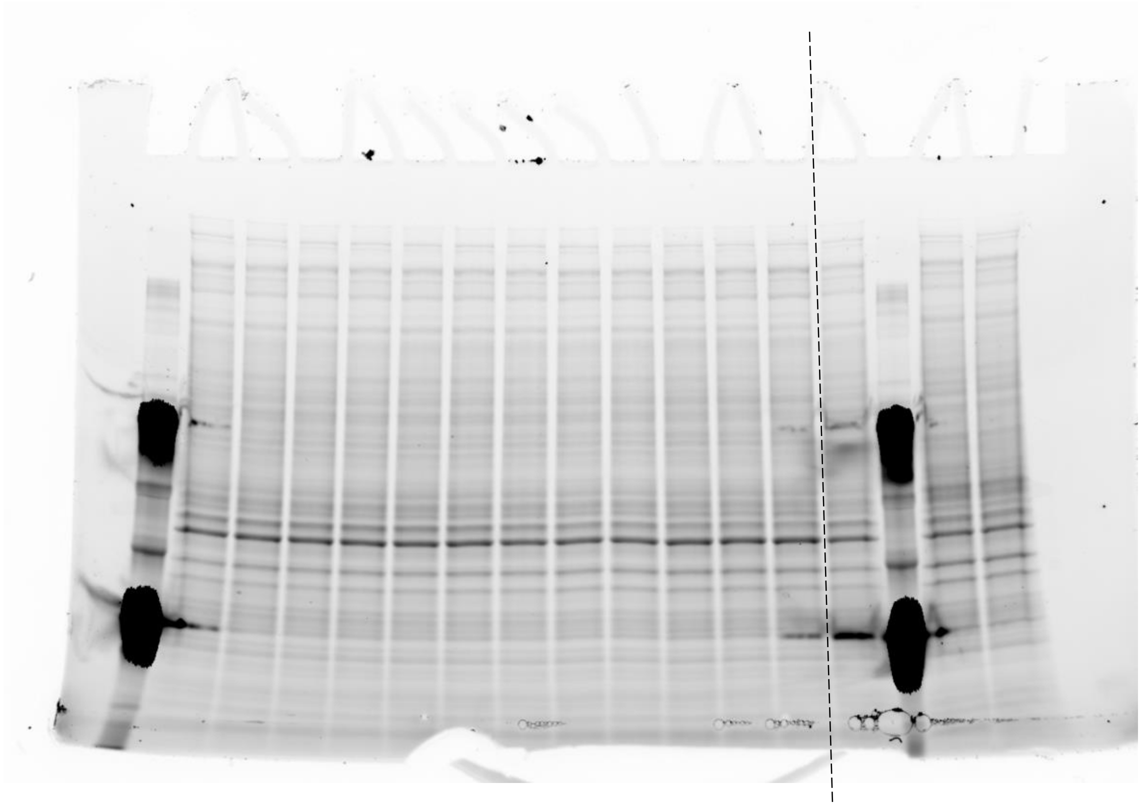

HUVEC

Replicate 2

HIF-3α2

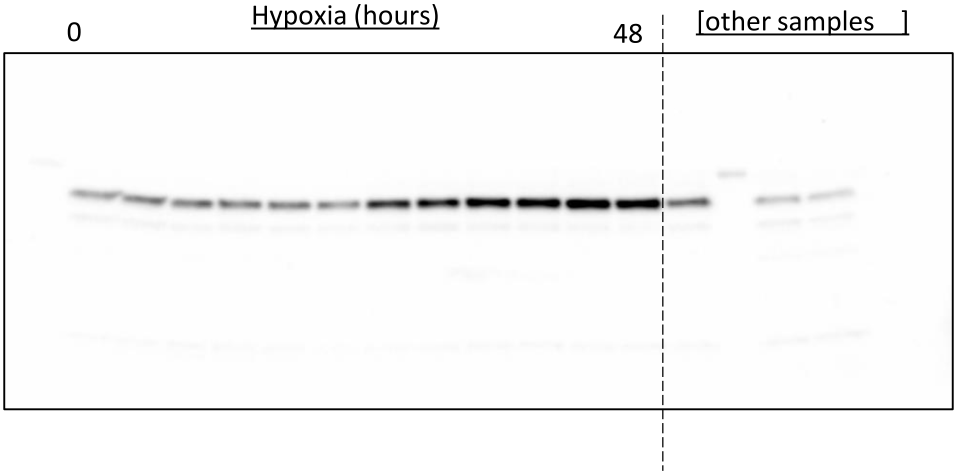

Actin β

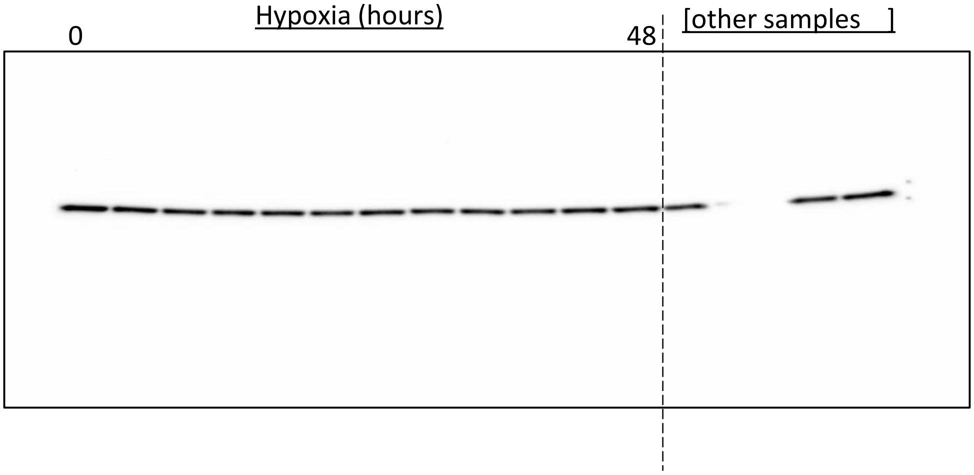

HUVEC

Replicate 3

HIF-3α2

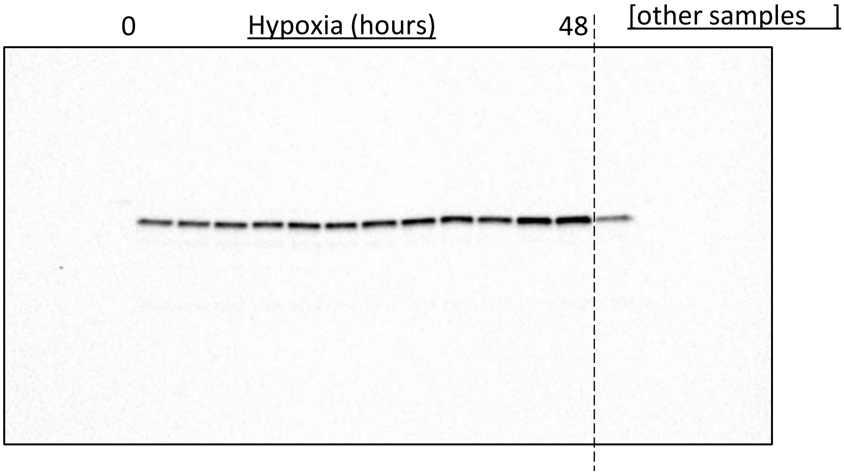

Actin β

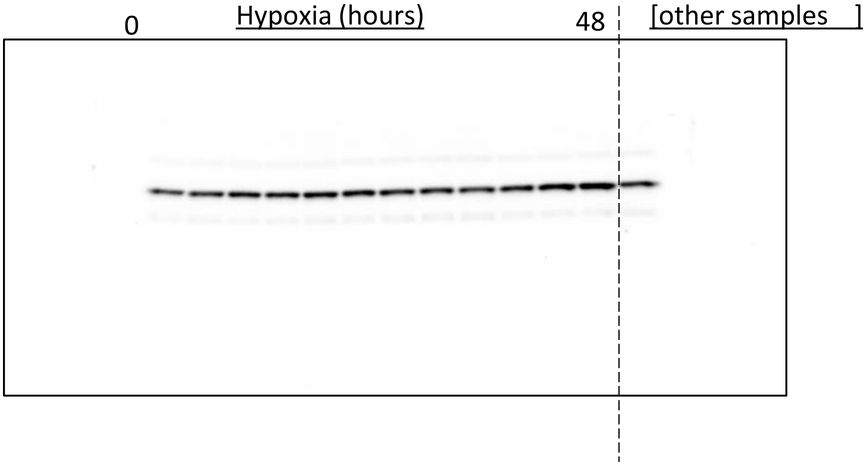

Total Protein

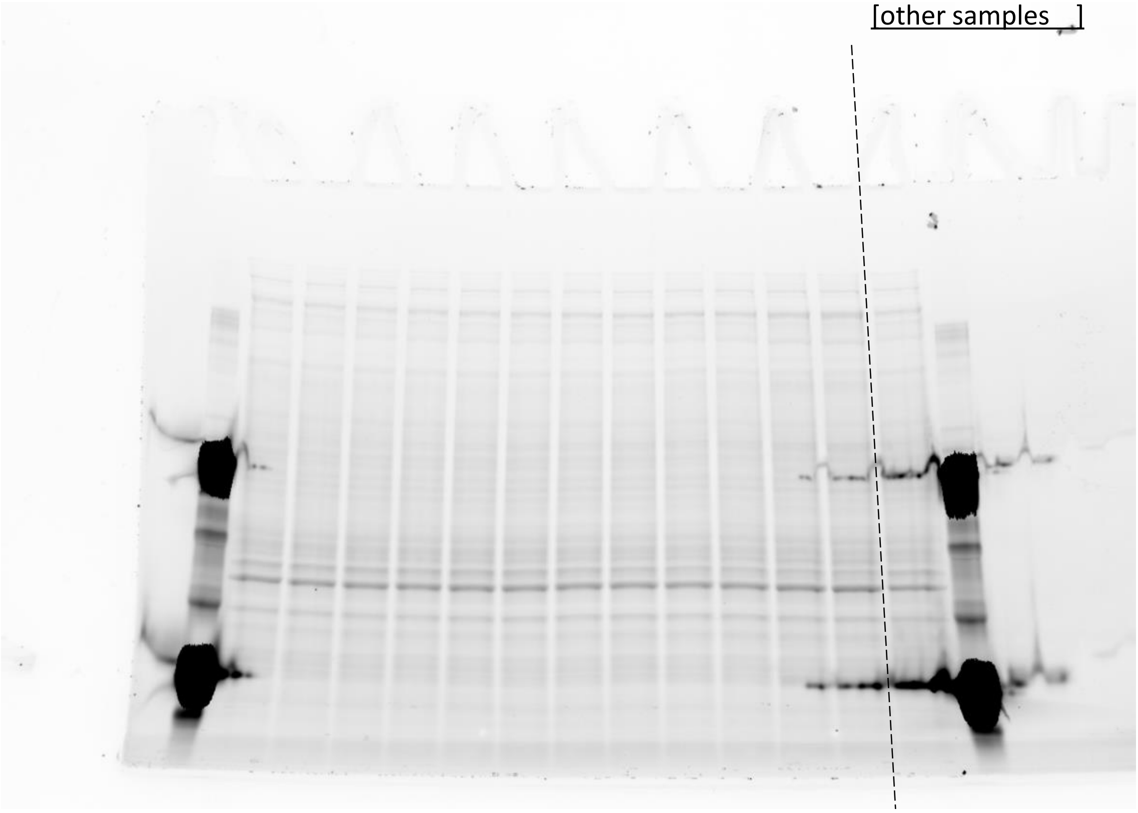

UtMVEC

Replicate 1

HIF-3α2

0 Hypoxia (hours) 48 [other samples]

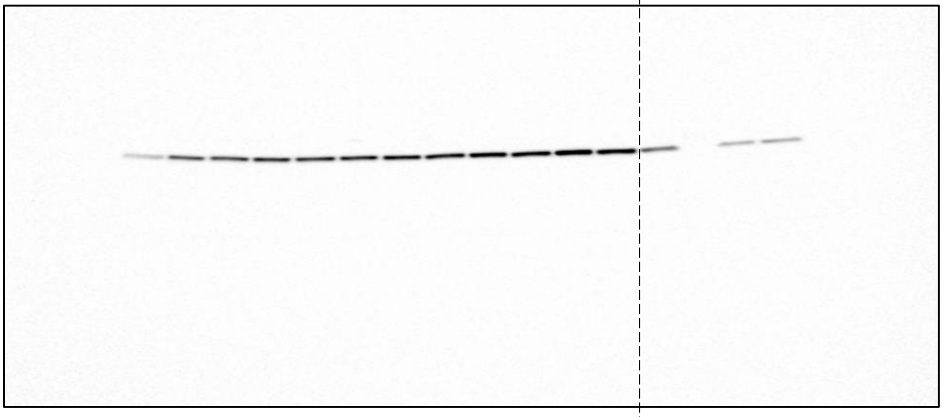

Total Protein

Hypoxia (hours) [other samples]

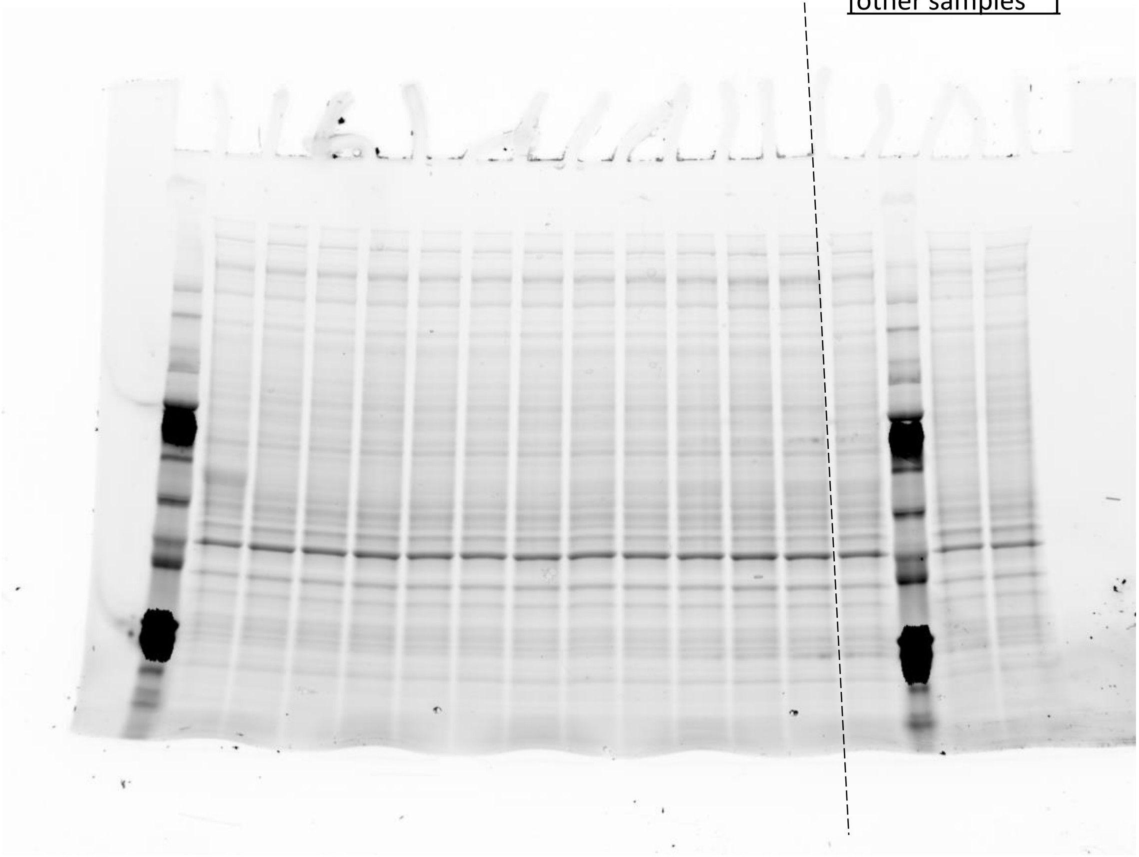

UtMVEC

Replicate 2

HIF-3α2

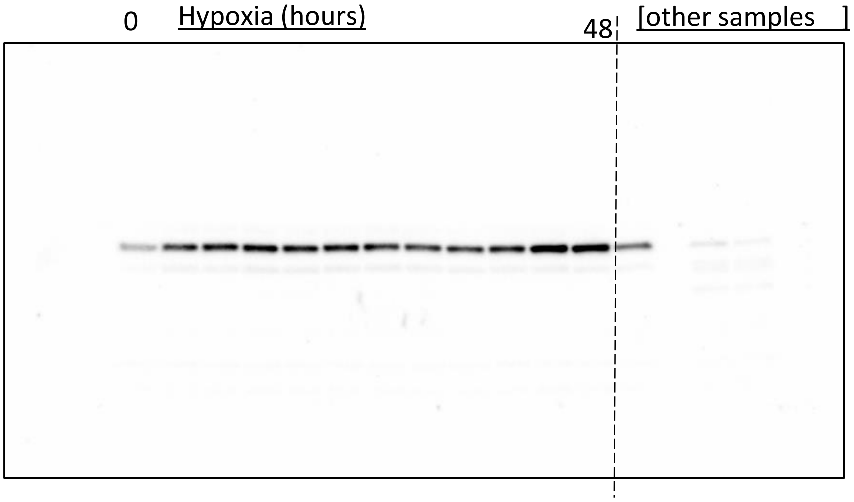

Total Protein

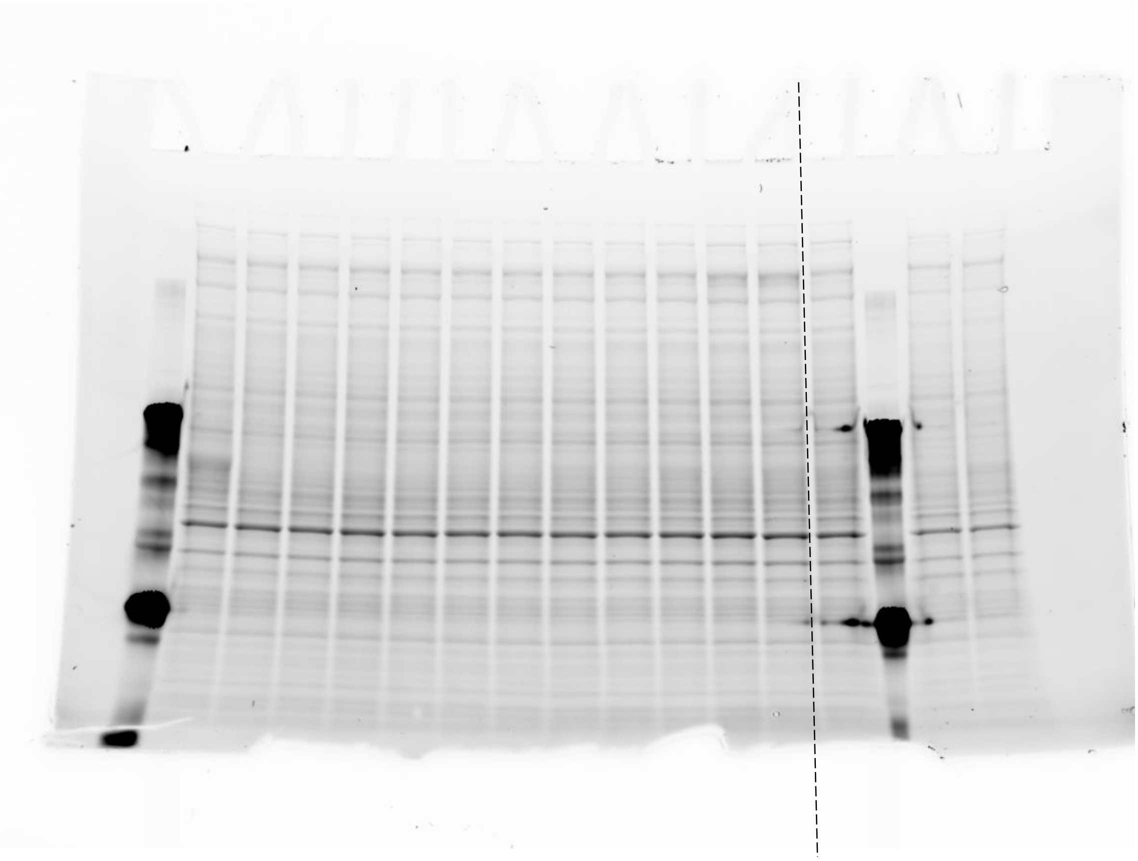

UtMVEC

Replicate 3

HIF-3α2 0 Hypoxia (hours) 48 [other samples]

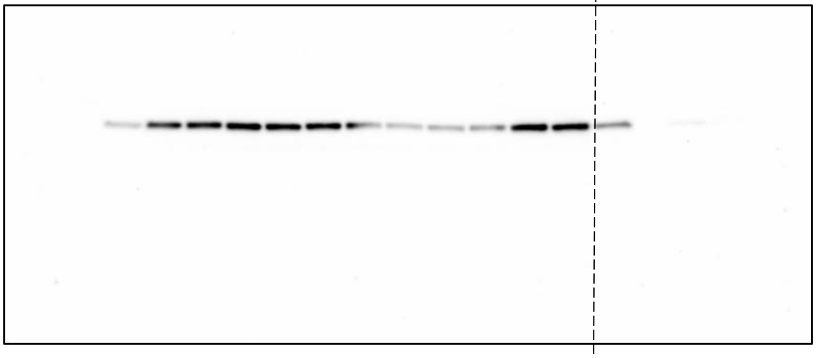

Actin β 0 Hypoxia (hours) 48 [other samples]

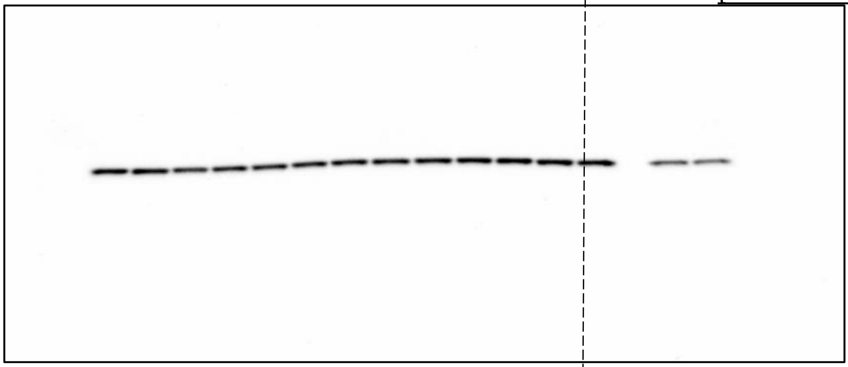

Total Protein

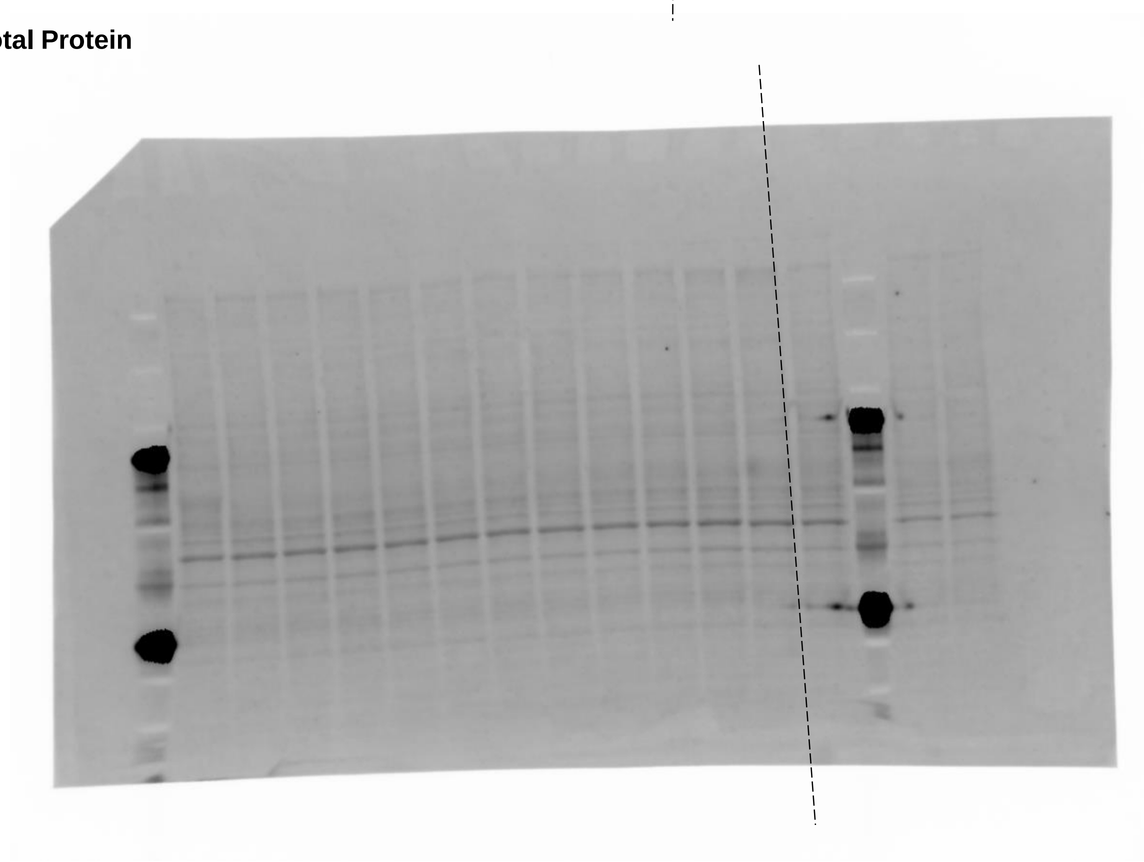

HPAEC

Replicate 1

HIF-3α2

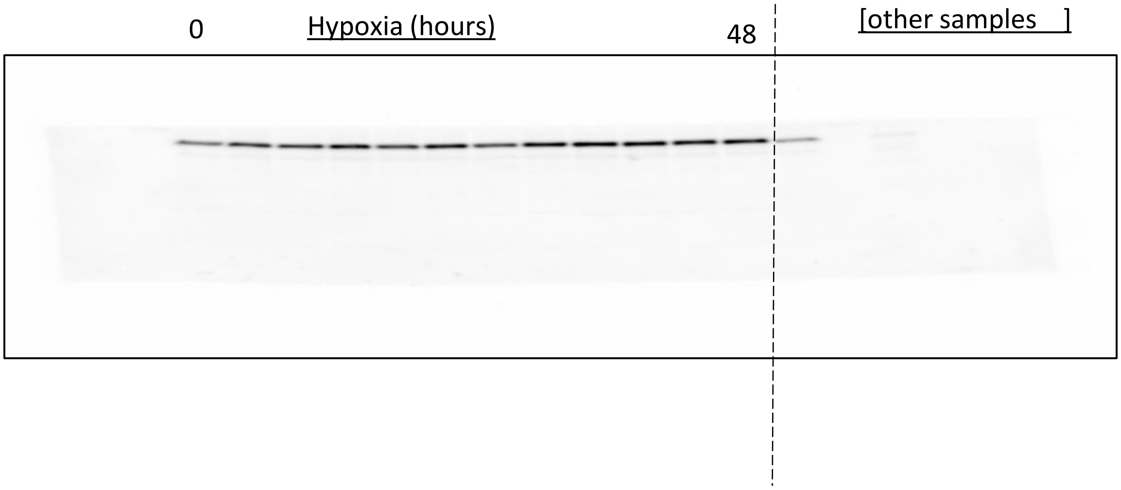

Actin β

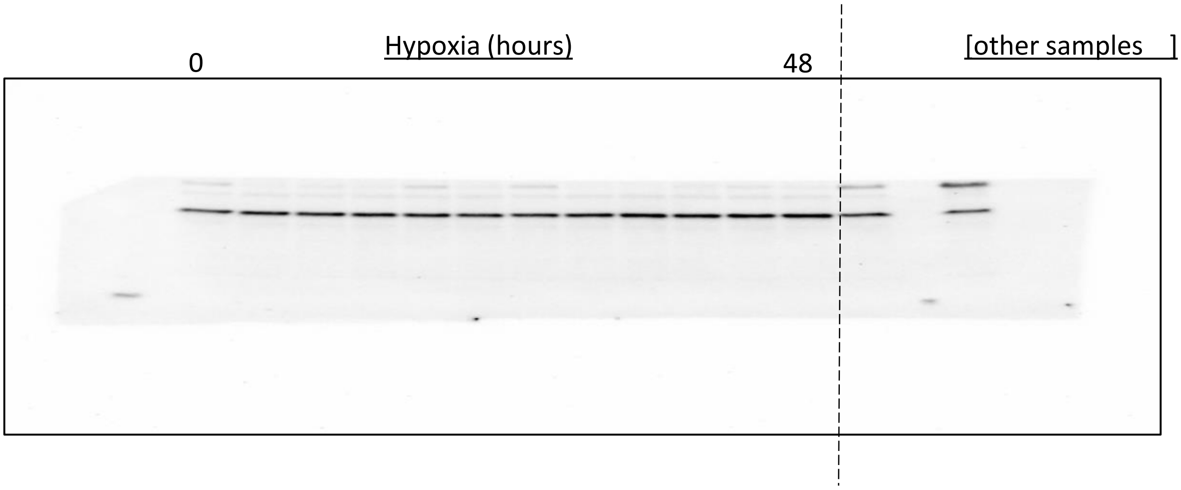

Total Protein

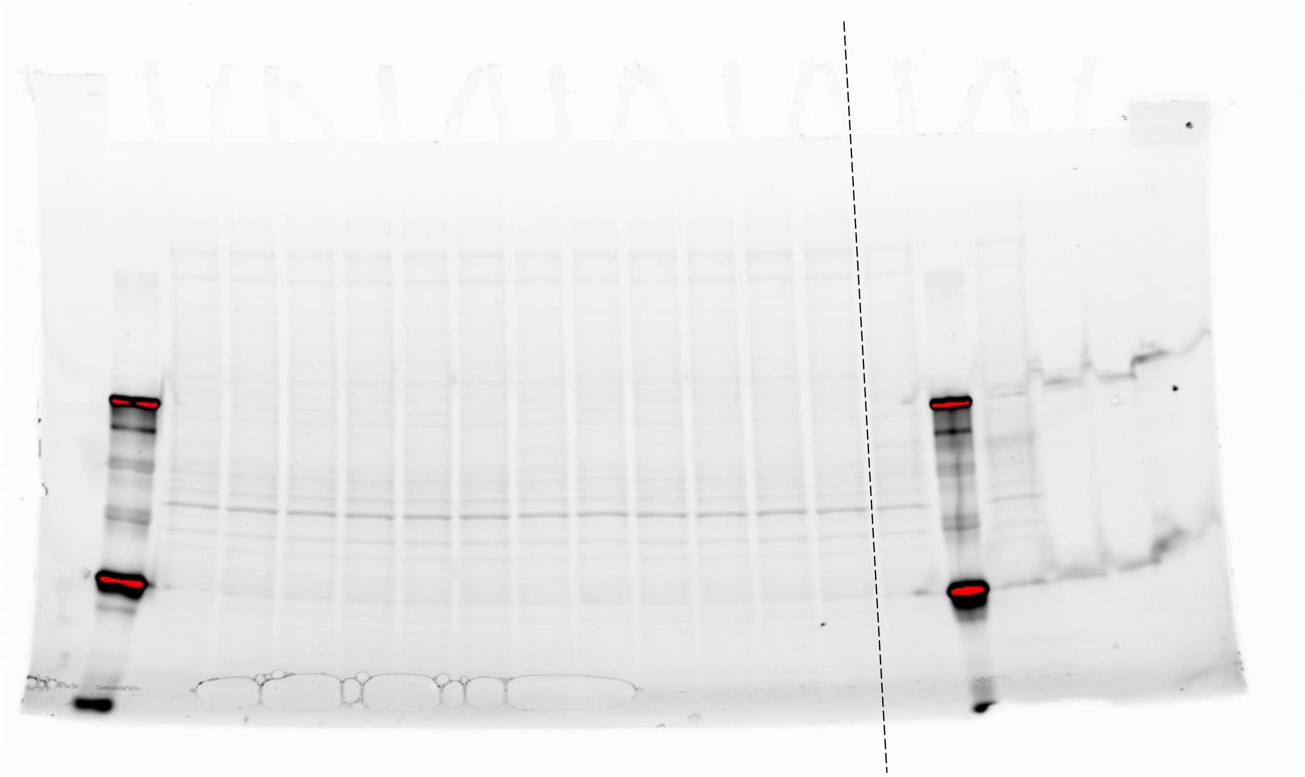

HPAEC

Replicate 2

HIF-3α2

0 Hypoxia (hours) 48 [other samples]

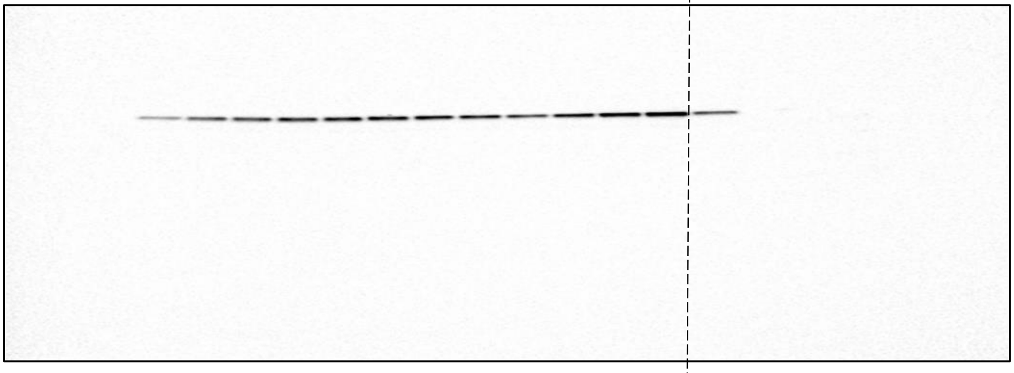

Actin β

0 Hypoxia (hours) 48 [other samples]

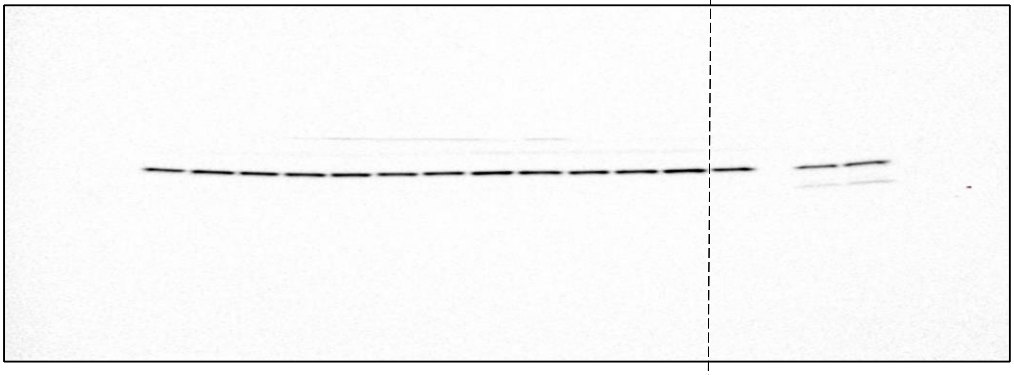

Total Protein

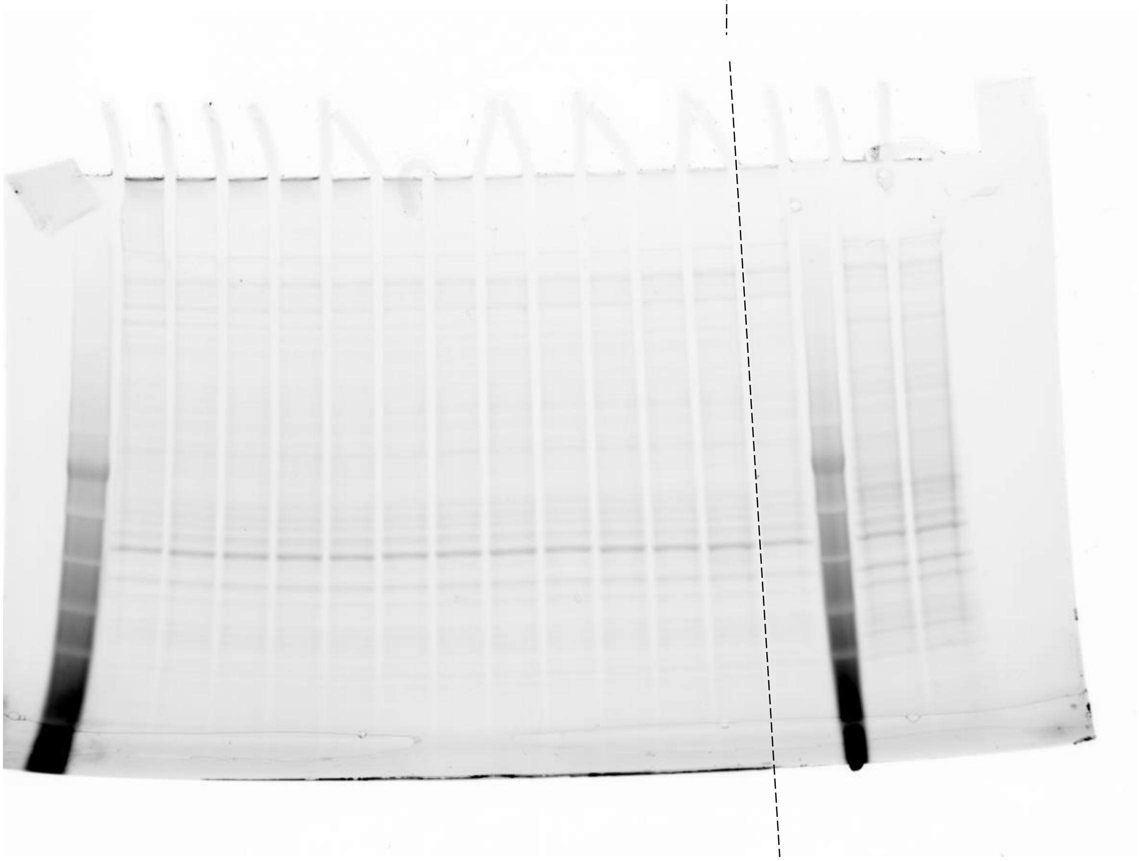

HPAEC

Replicate 3

HIF-3α2

0 Hypoxia (hours) 48

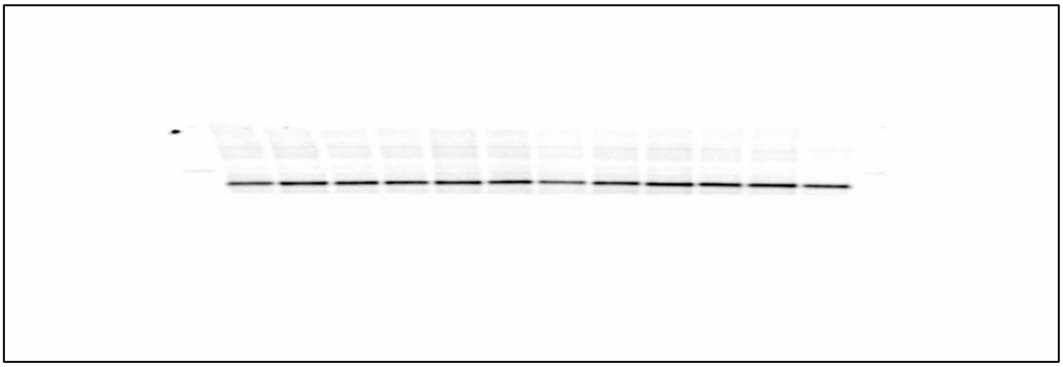

Actin β

0 Hypoxia (hours) 48

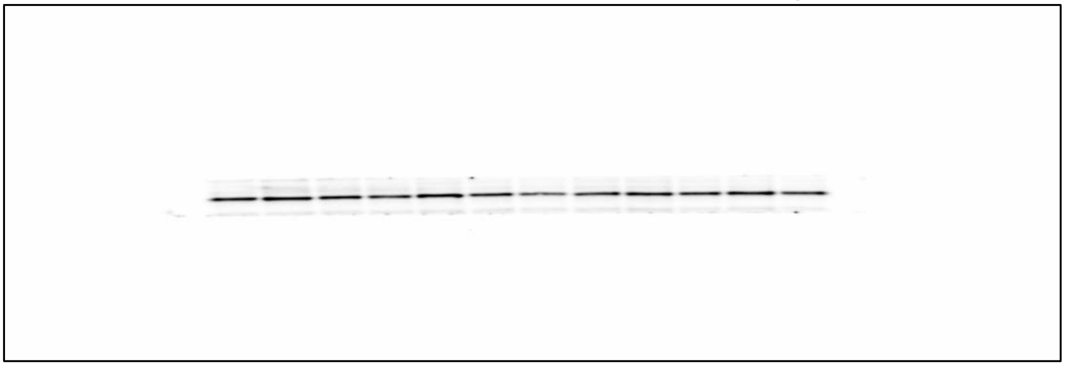

Total Protein

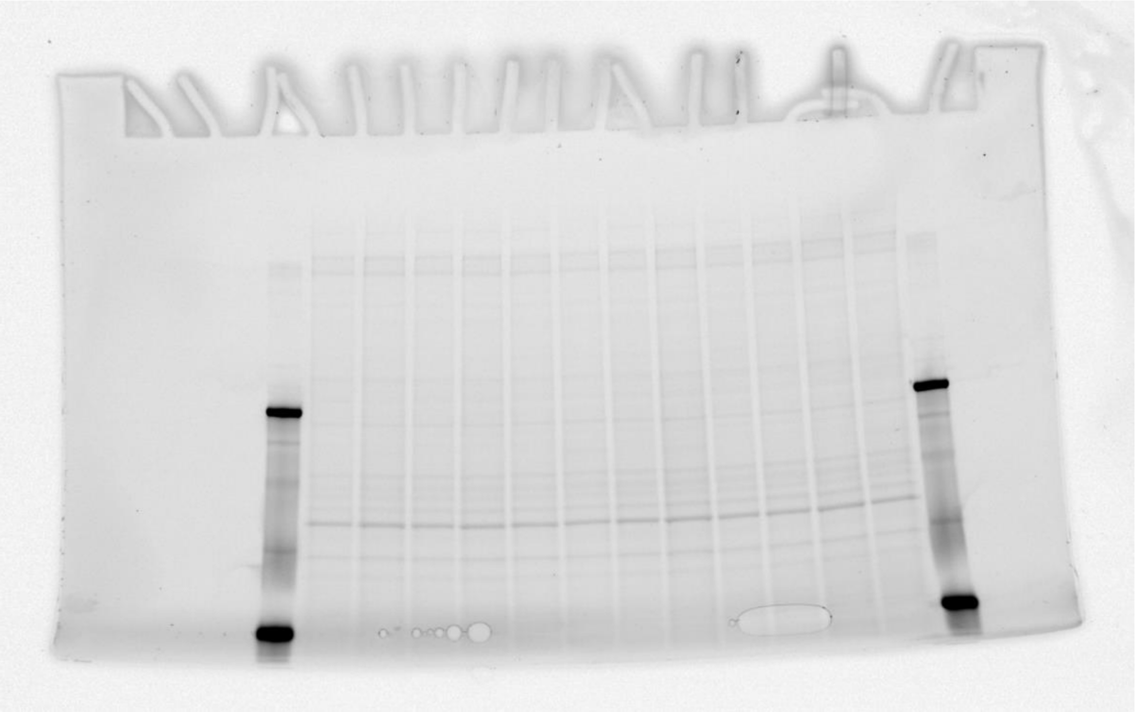

HCAEC

Replicate 1

HIF-3α2

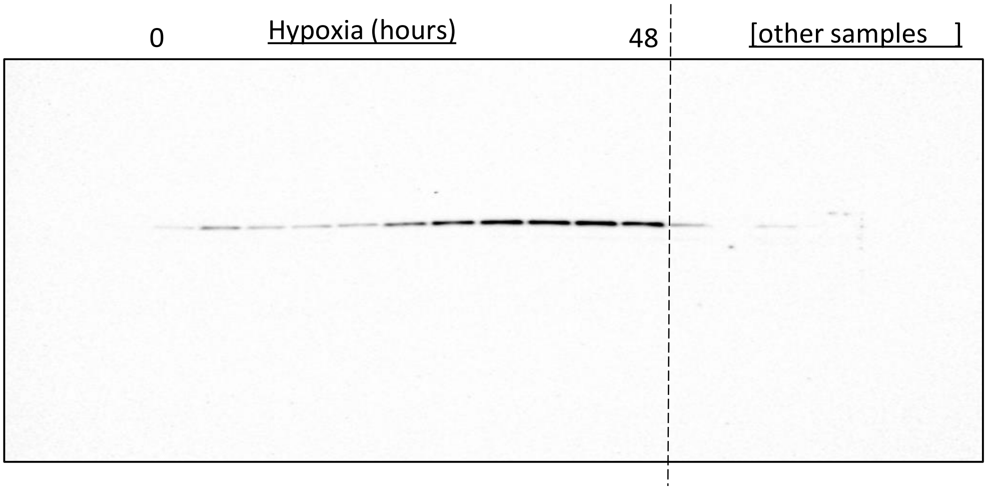

Actin β

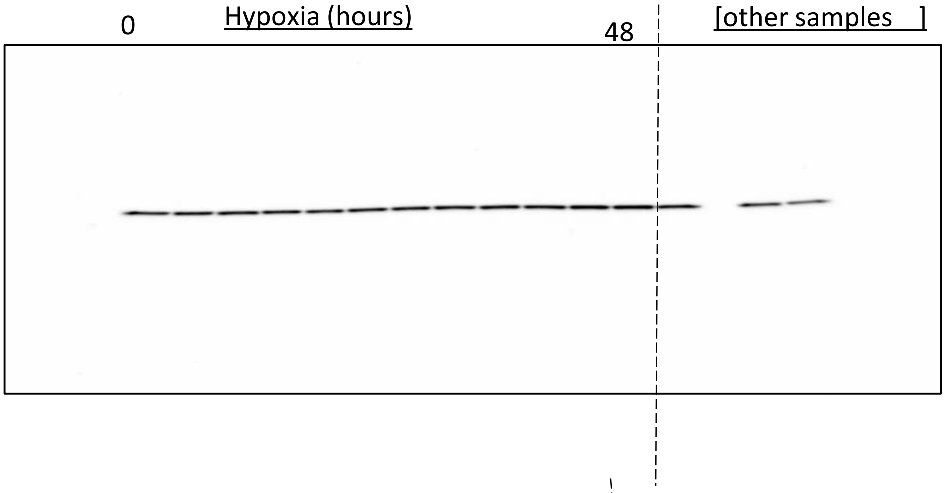

Total Protein

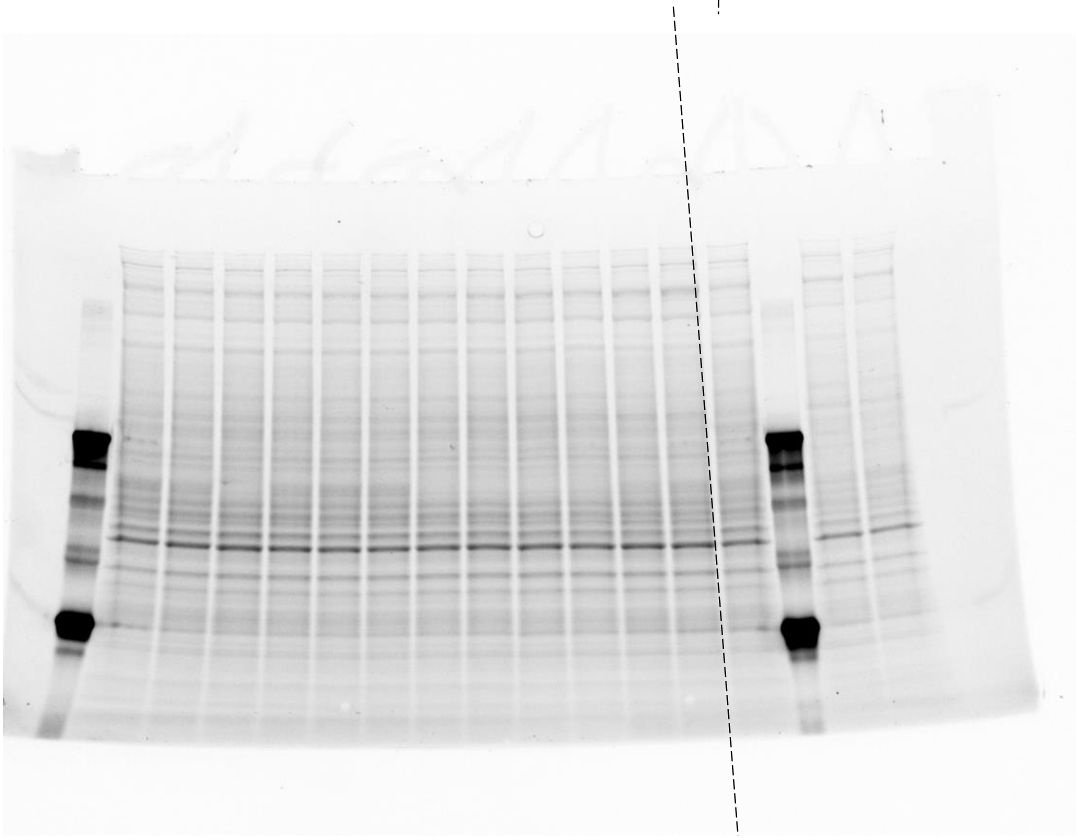

HCAEC

Replicate 2

HIF-3α2

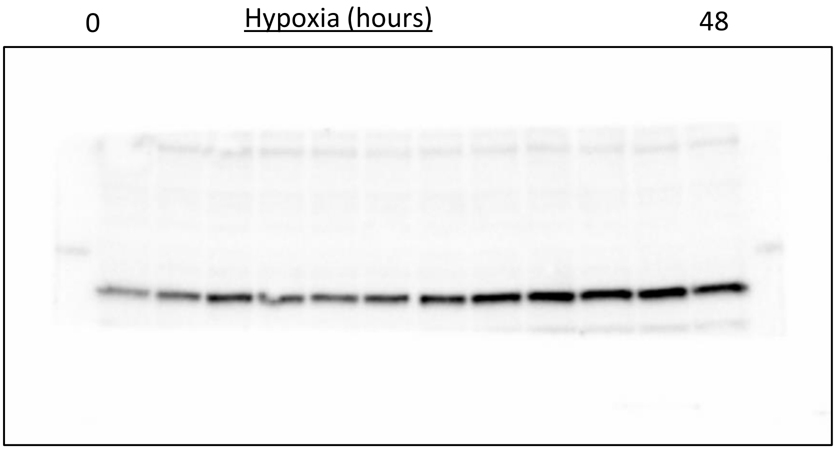

Actin β

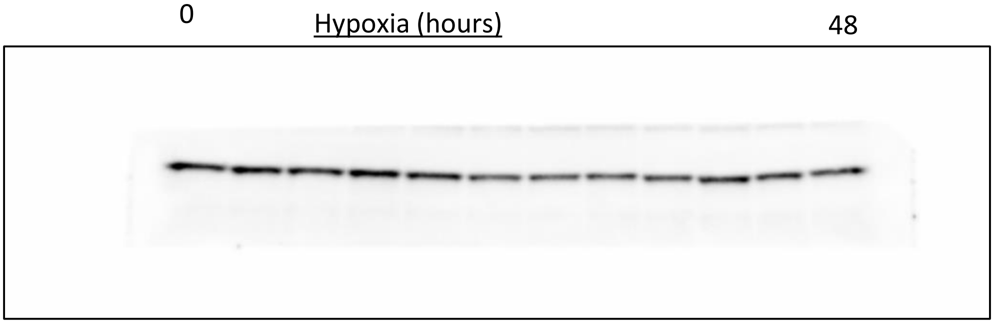

Total Protein

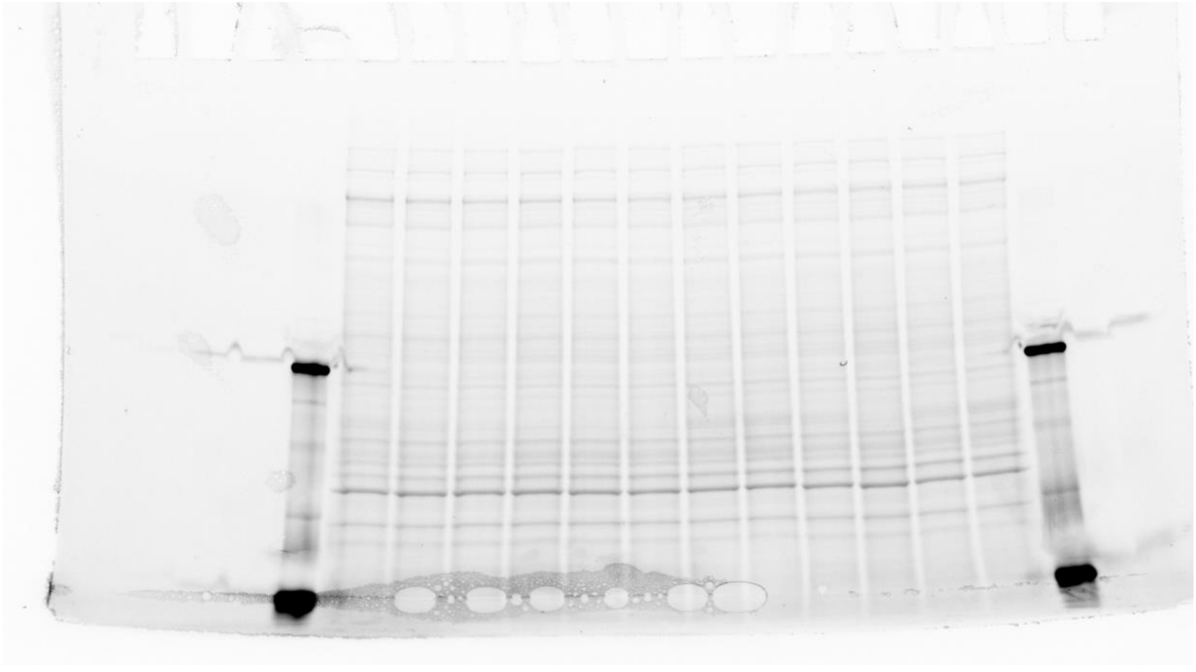

HCAEC

Replicate 3

HIF-3α2

0 Hypoxia (hours) 48

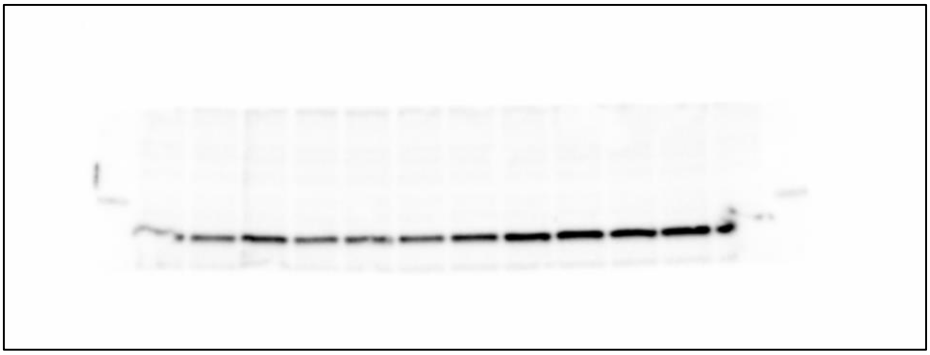

Actin β

0 Hypoxia (hours) 48

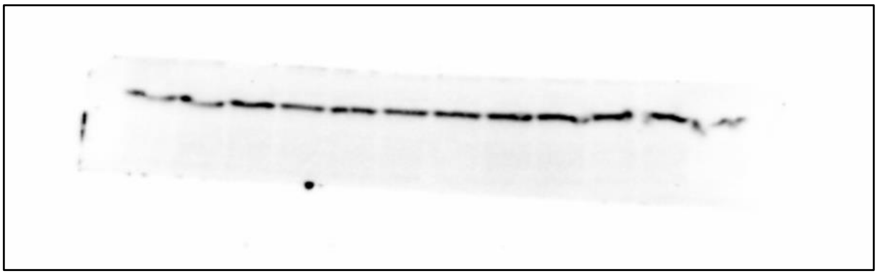

Total Protein

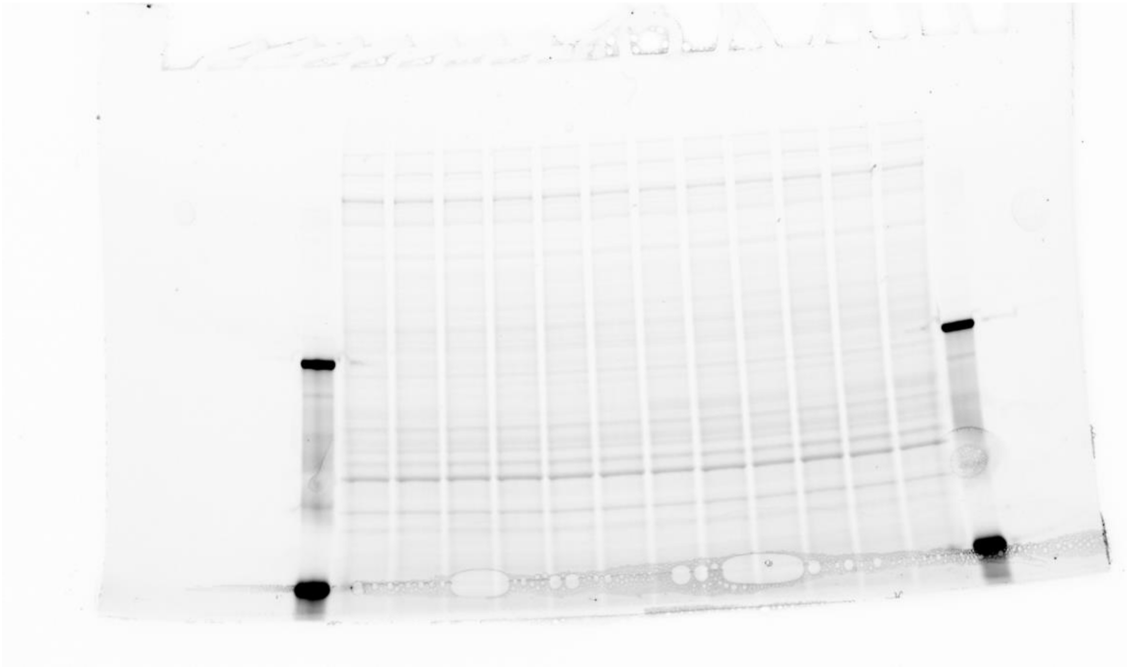

HIAEC

Replicate 1

HIF-3α2

0 Hypoxia (hours) 48 [other samples]

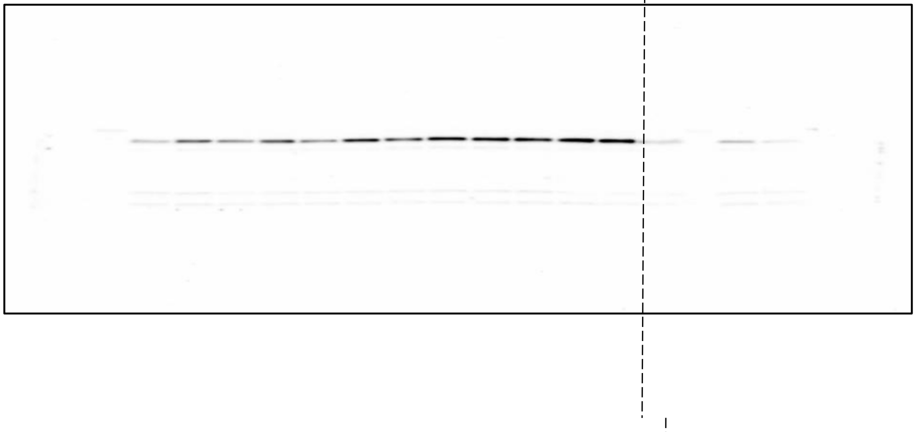

Actin β

0 Hypoxia (hours) 48 [other samples]

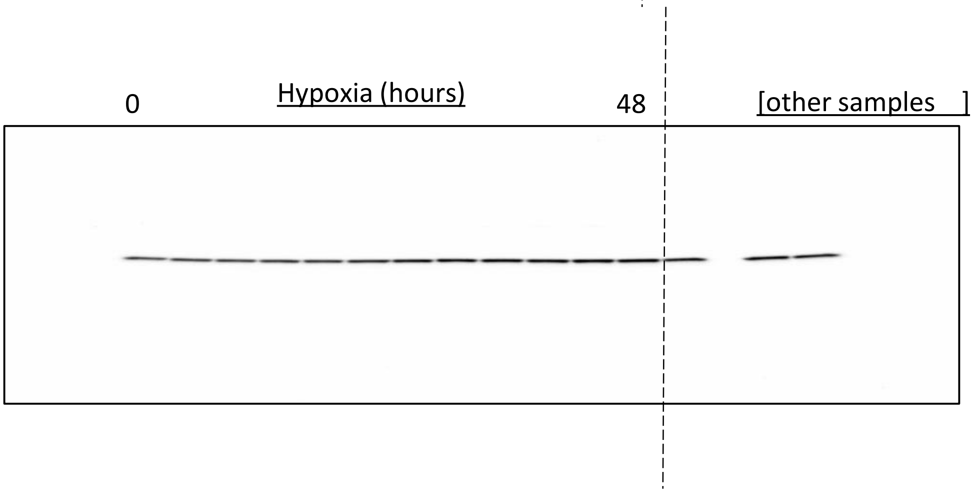

Total Protein

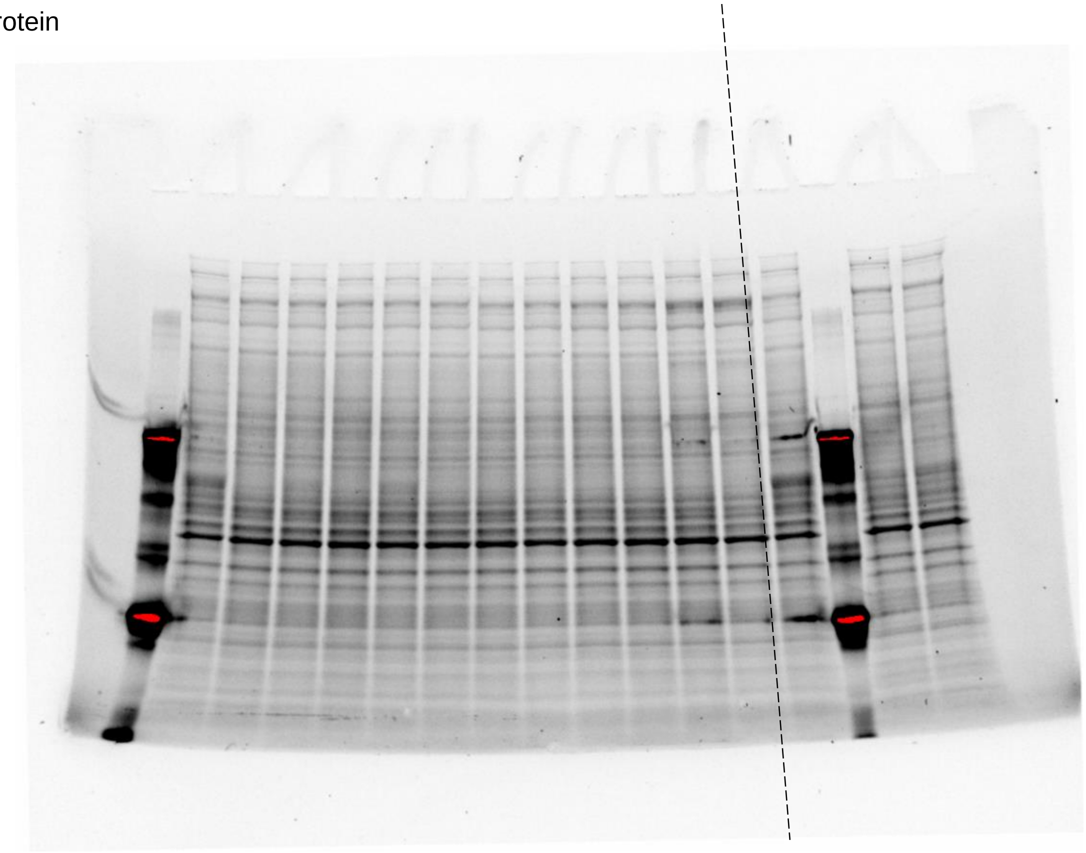

HIAEC

Replicate 2

HIF-3α2

Hypoxia (hours)

0 48

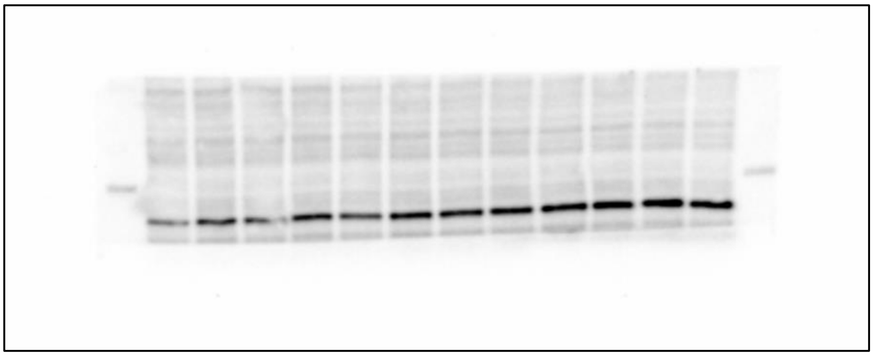

Actin β

Hypoxia (hours)

0 48

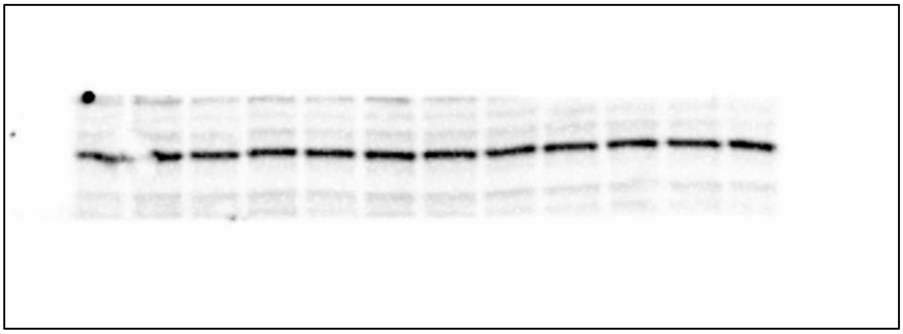

Total Protein

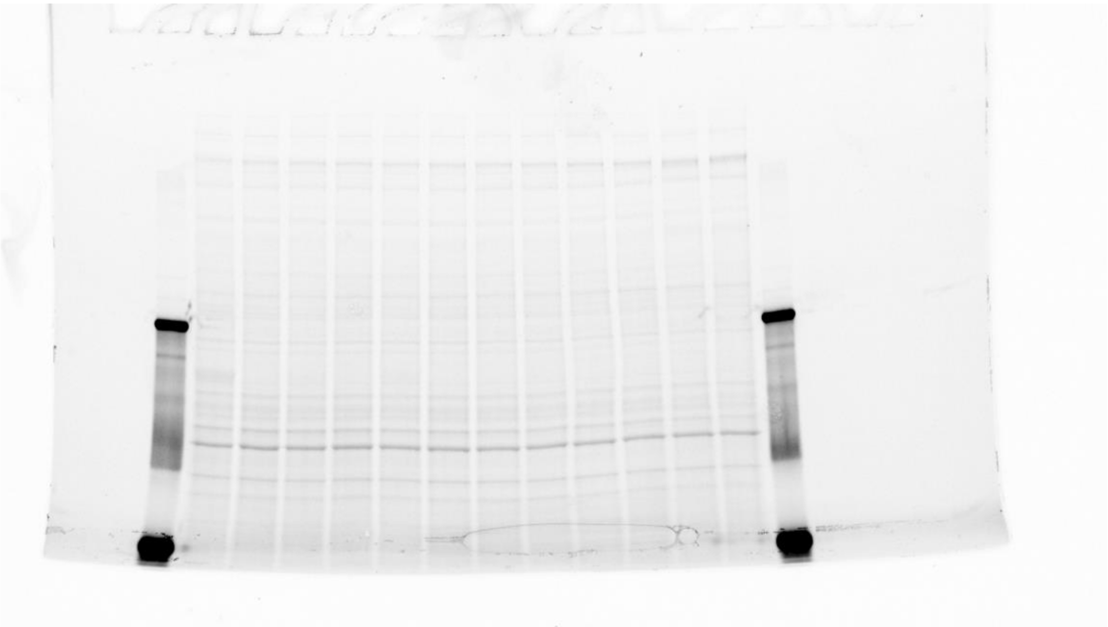

HIAEC

Replicate 3

HIF-3α2

0 Hypoxia (hours) 48

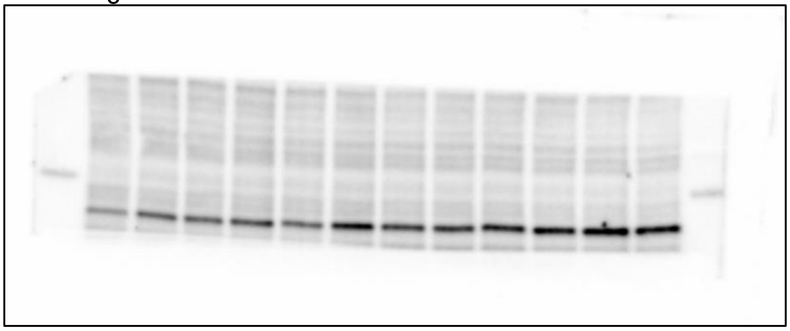

Actin β

0 Hypoxia (hours) 48

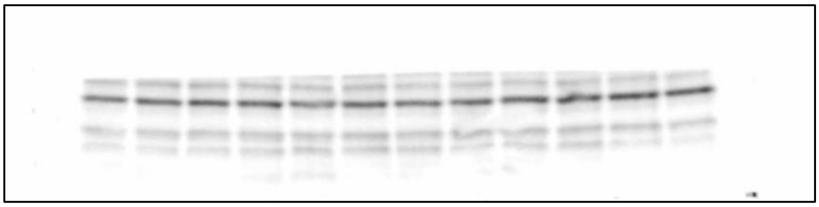

Total Protein

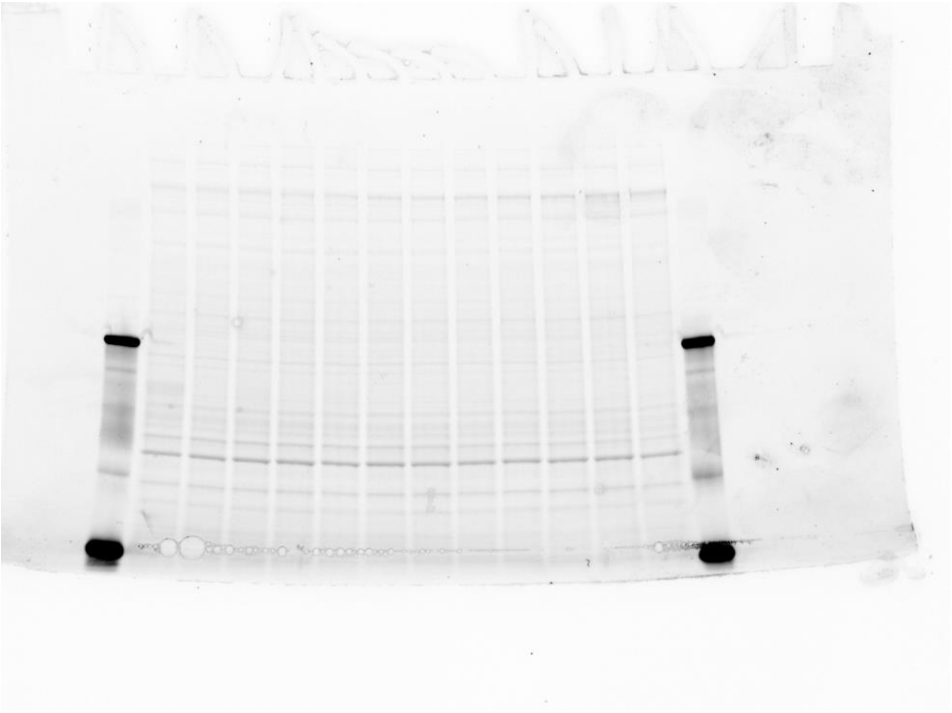

HPASMC

Replicate 1

HIF-3α2

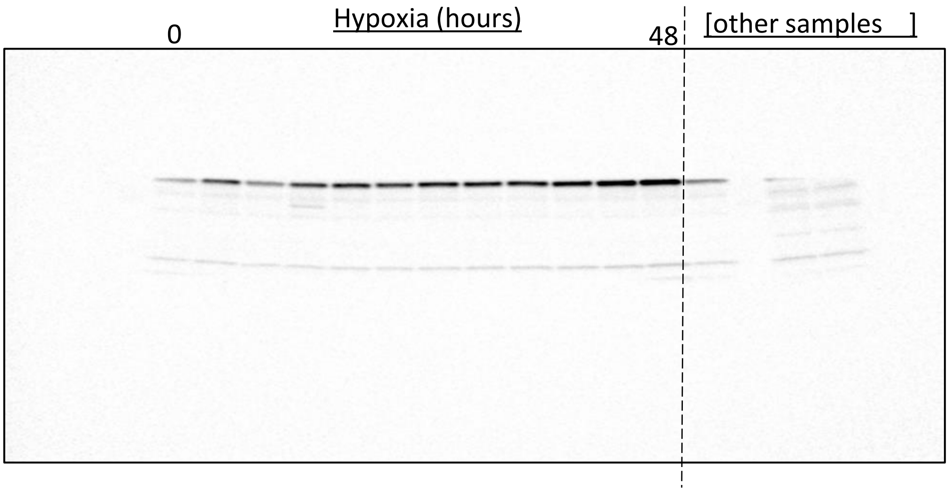

Total Protein

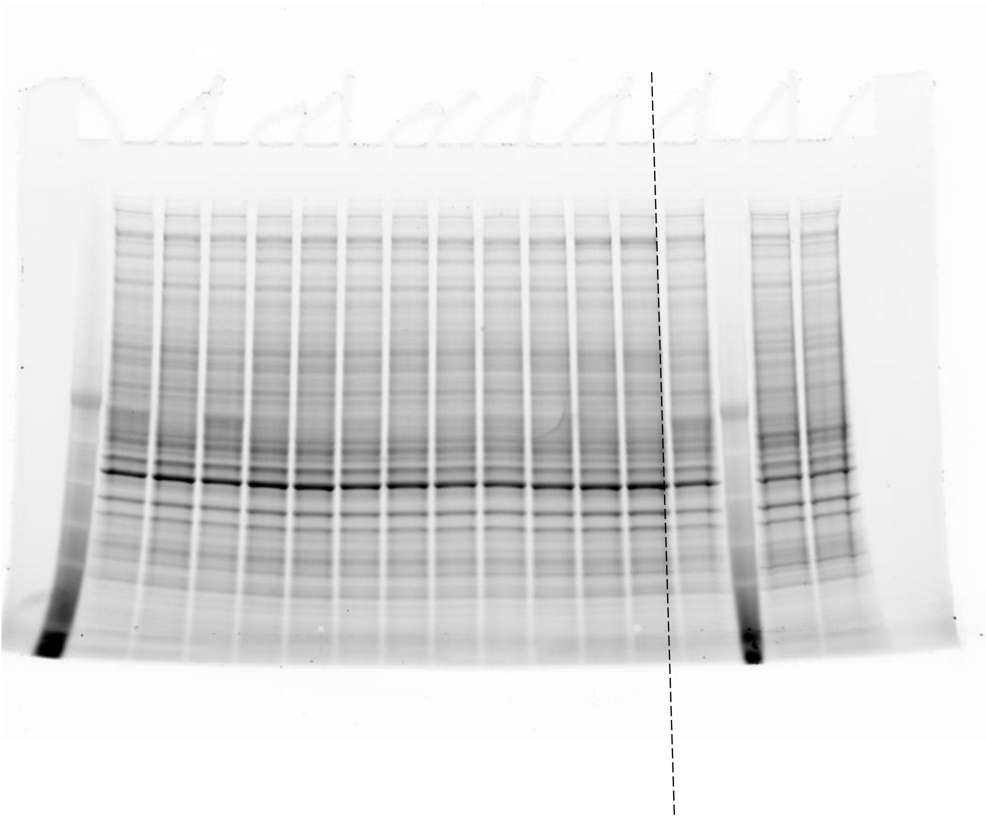

HPASMC

Replicate 2

HIF-3α2

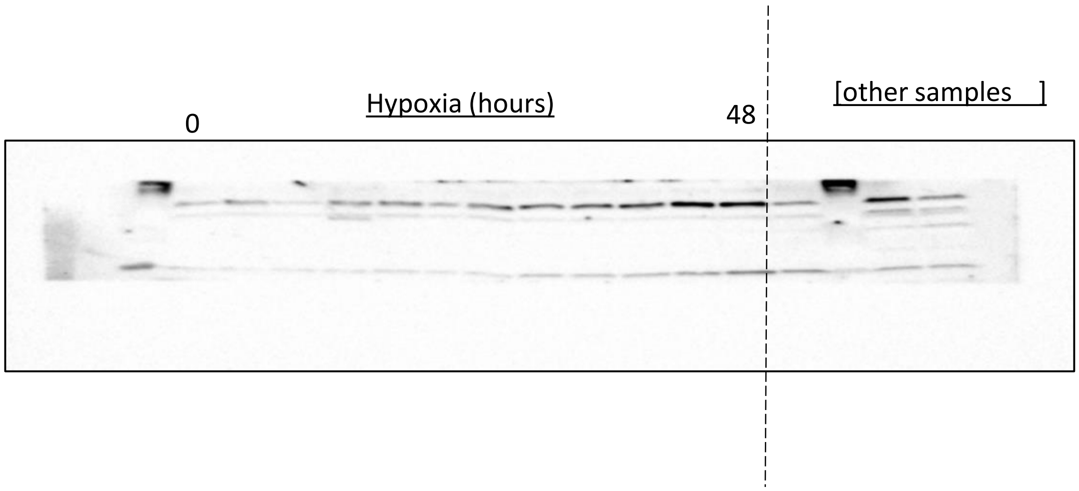

Actin β

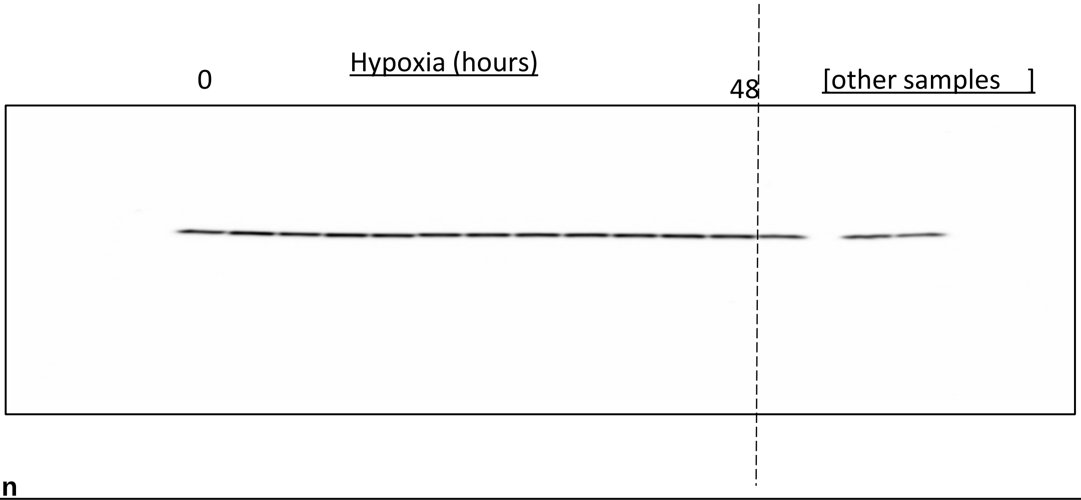

Total Protein

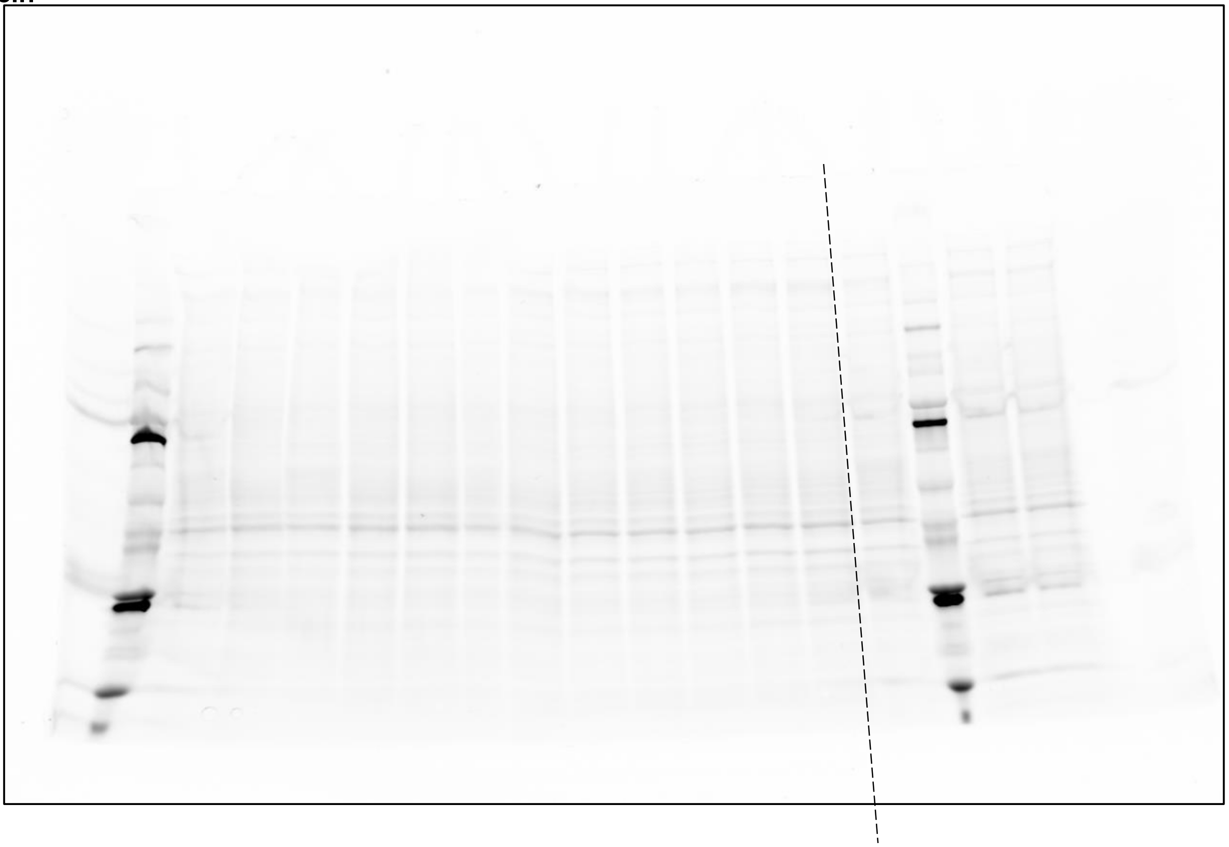

HPASMC

Replicate 3

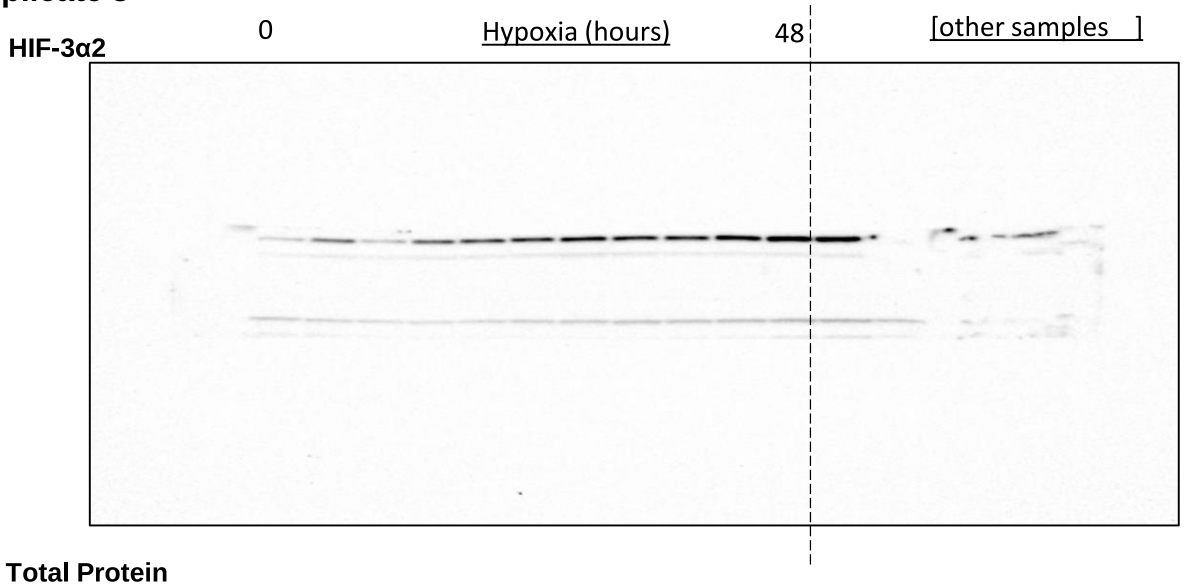

Total Protein

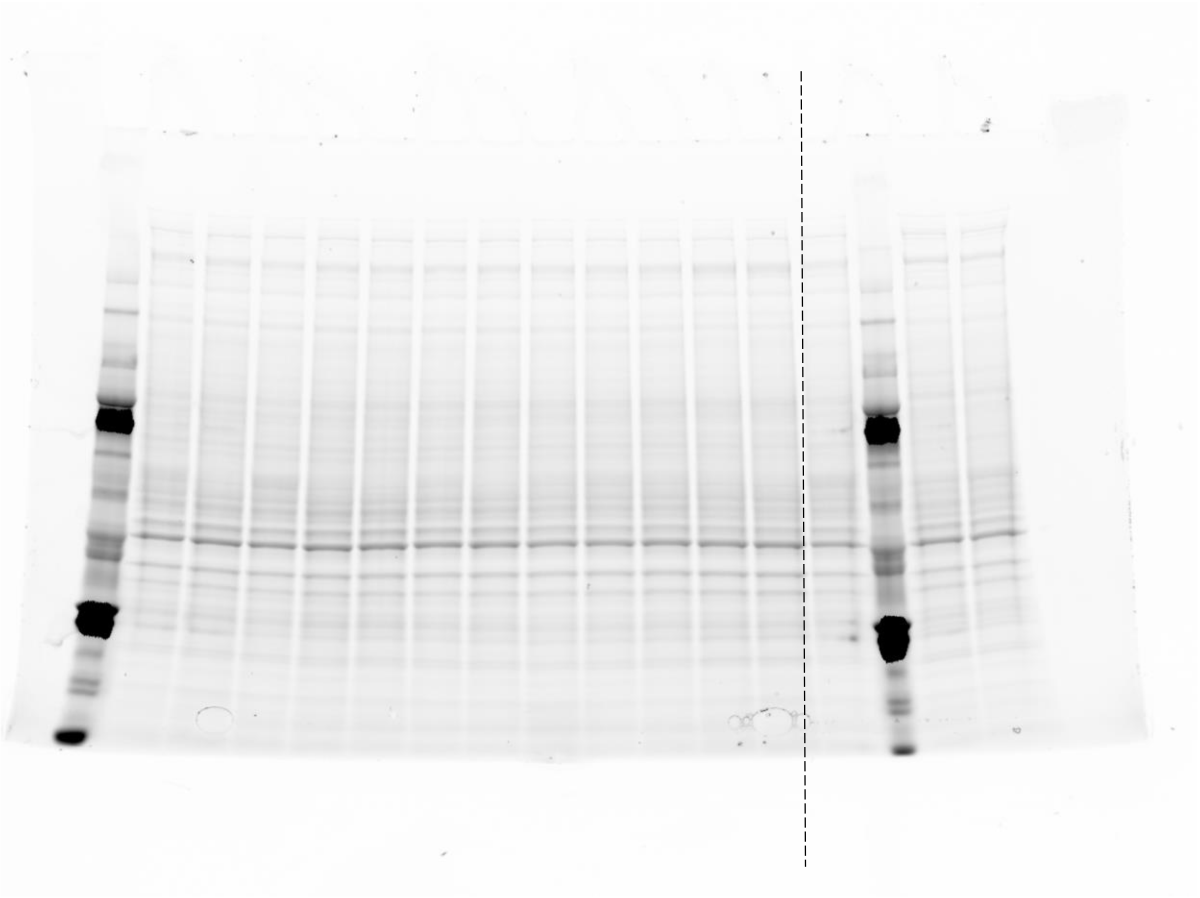

HMVEC-D

Replicate 1

HIF-3α2

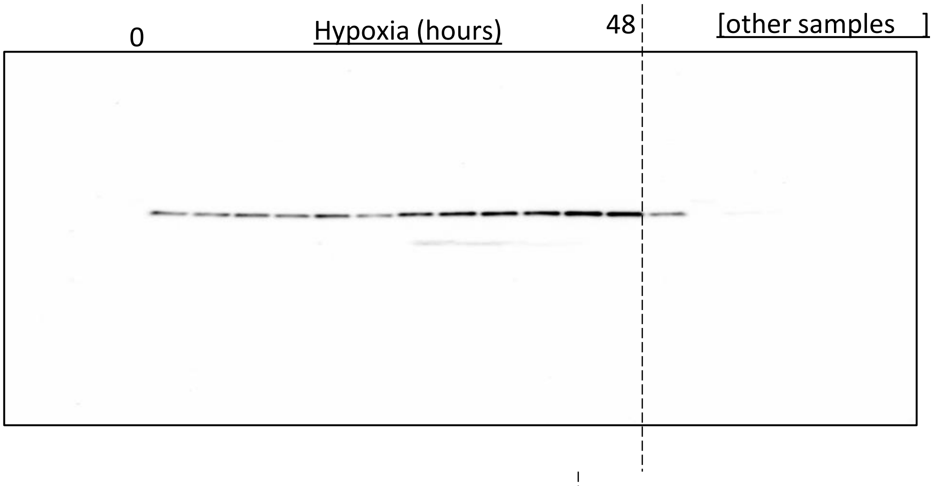

Actin β

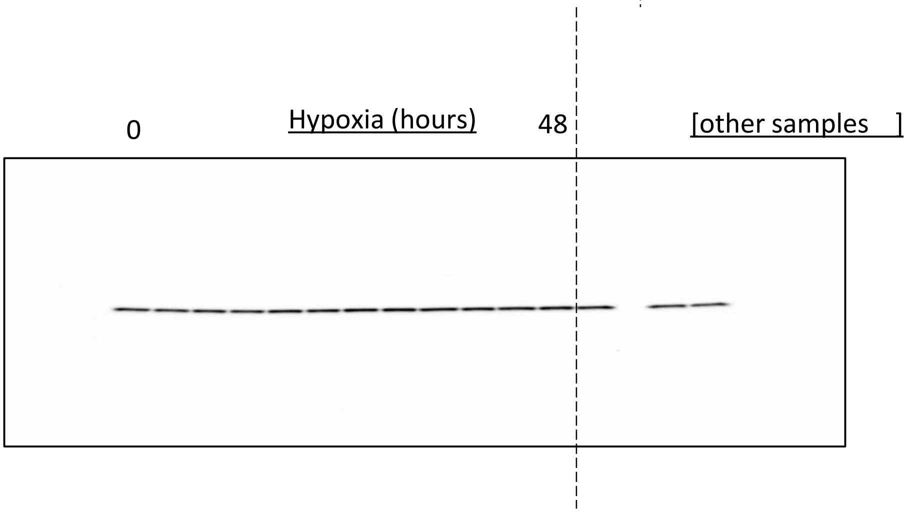

Total Protein

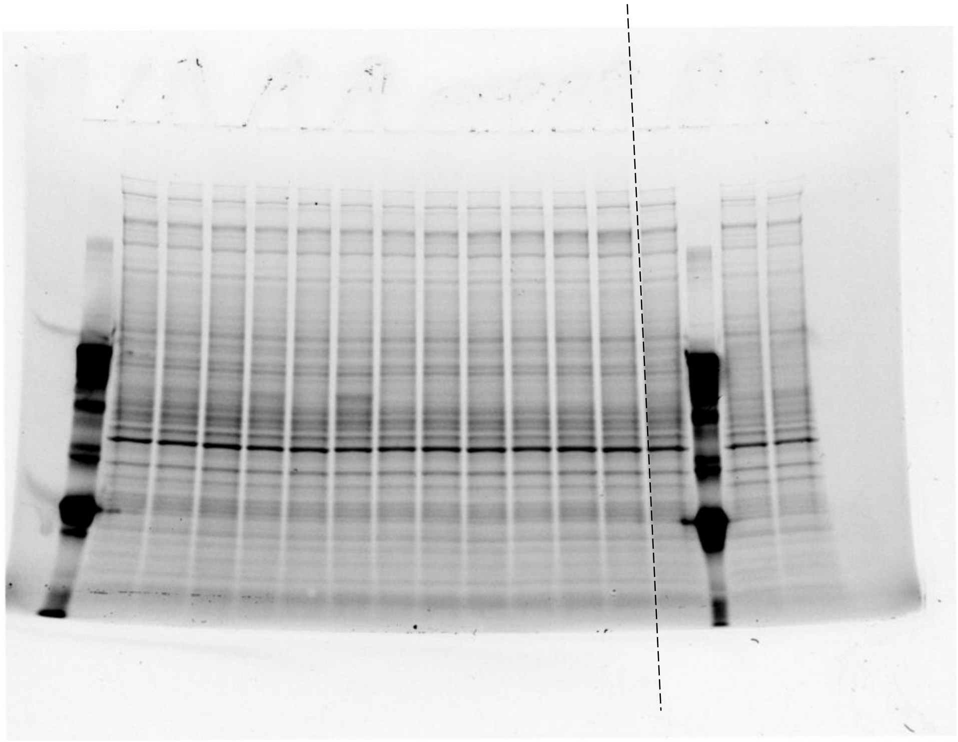

HMVEC-D

Replicate 2

HIF-3α2

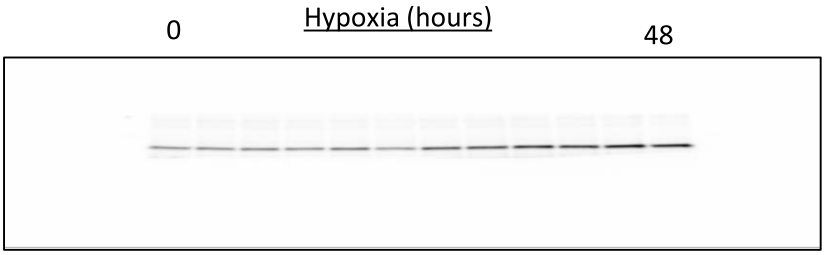

Actin β

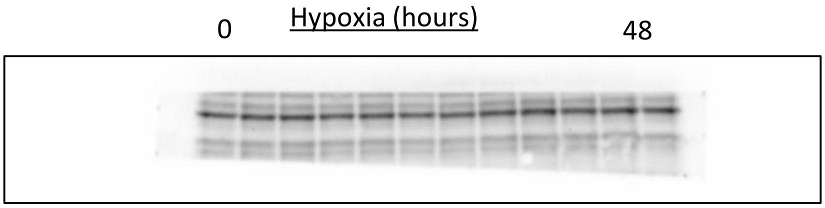

Total Protein

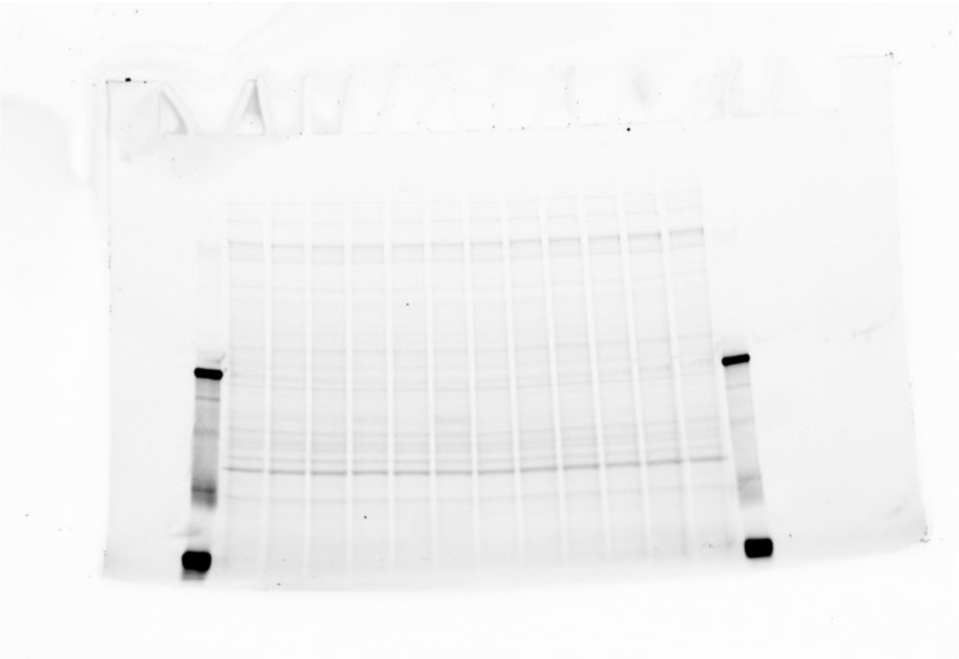

HMVEC-D

Replicate 3

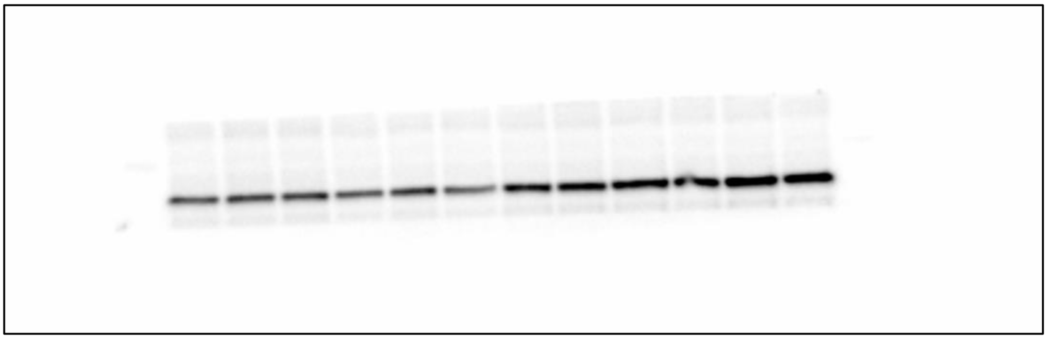

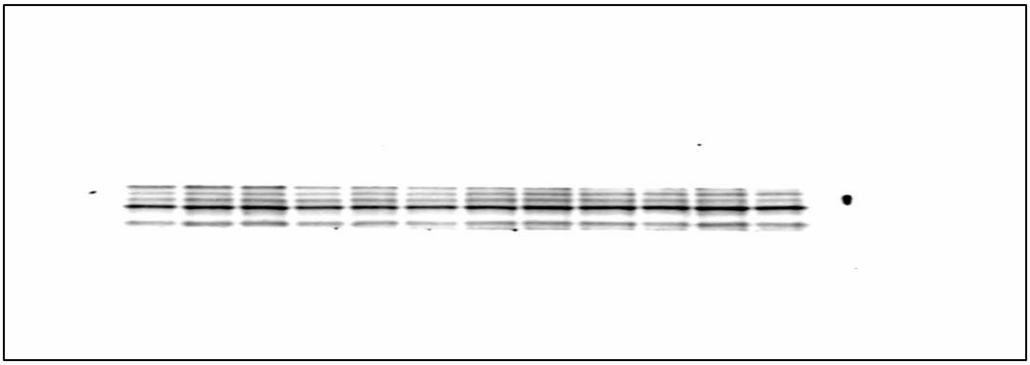

Total Protein

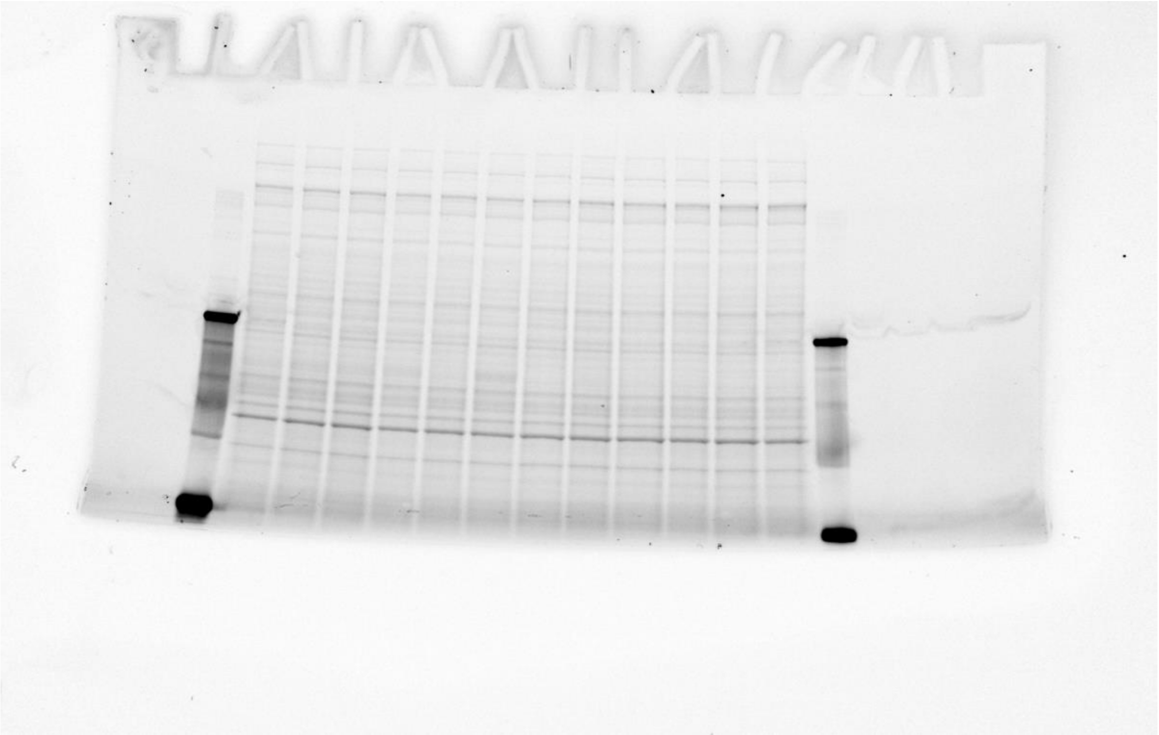

HMVEC-L

Replicate 1

HIF-3α2

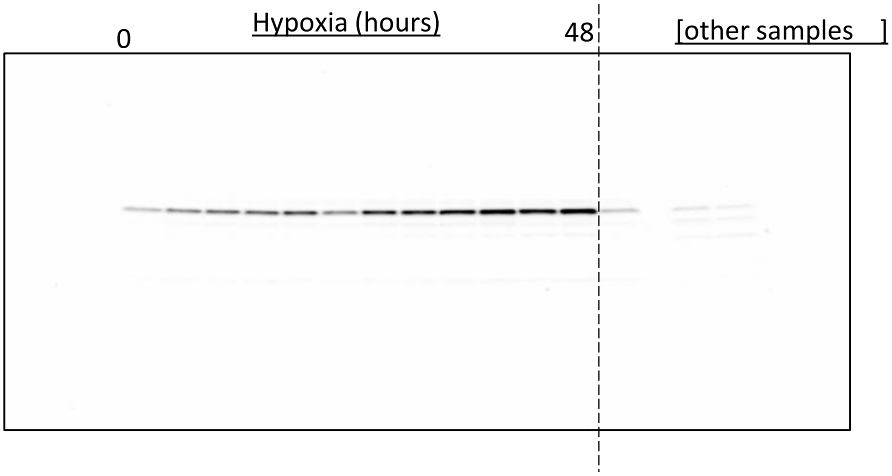

Actin β

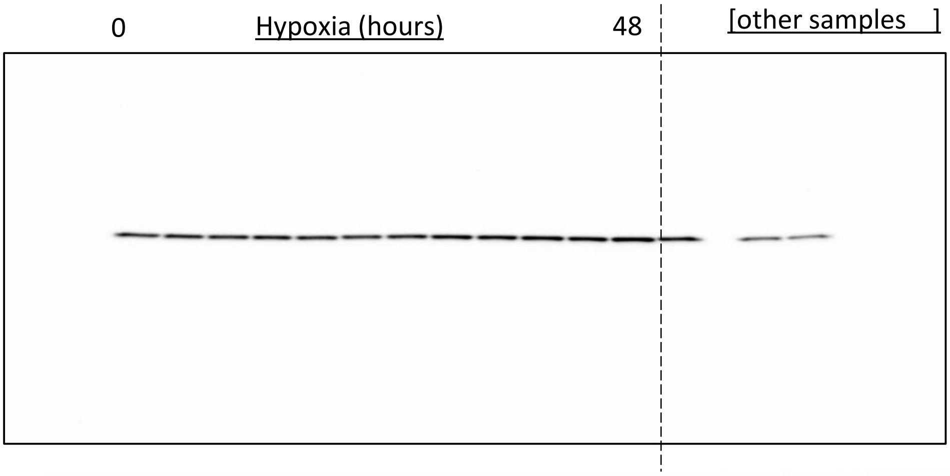

Total Protein

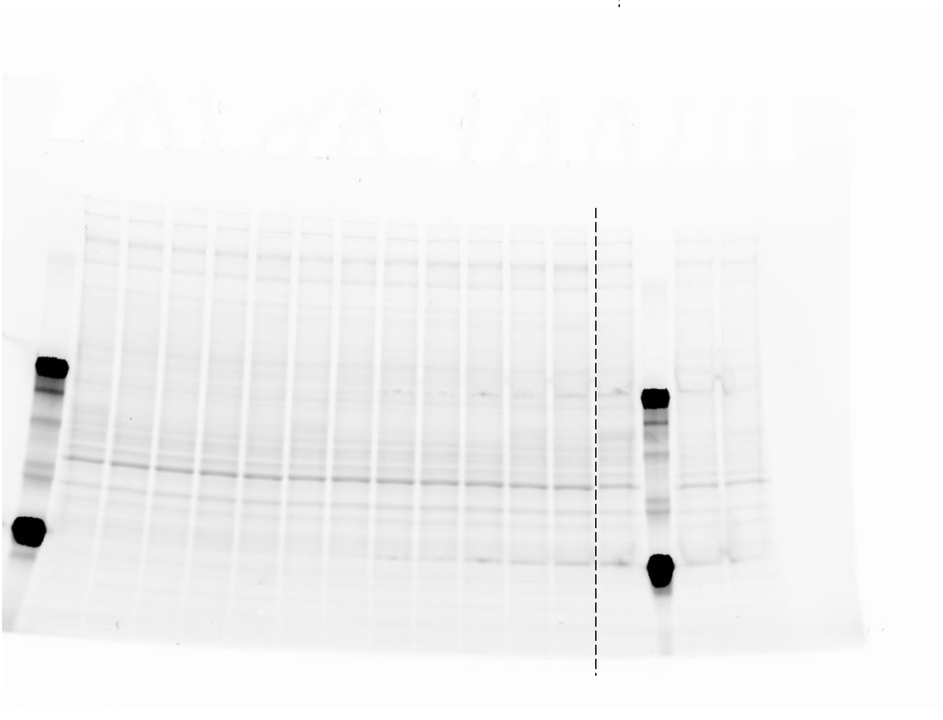

HMVEC-L

Replicate 2

HIF-3α2

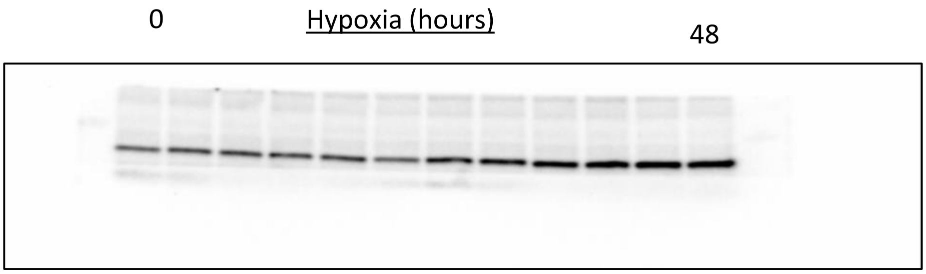

Total Protein

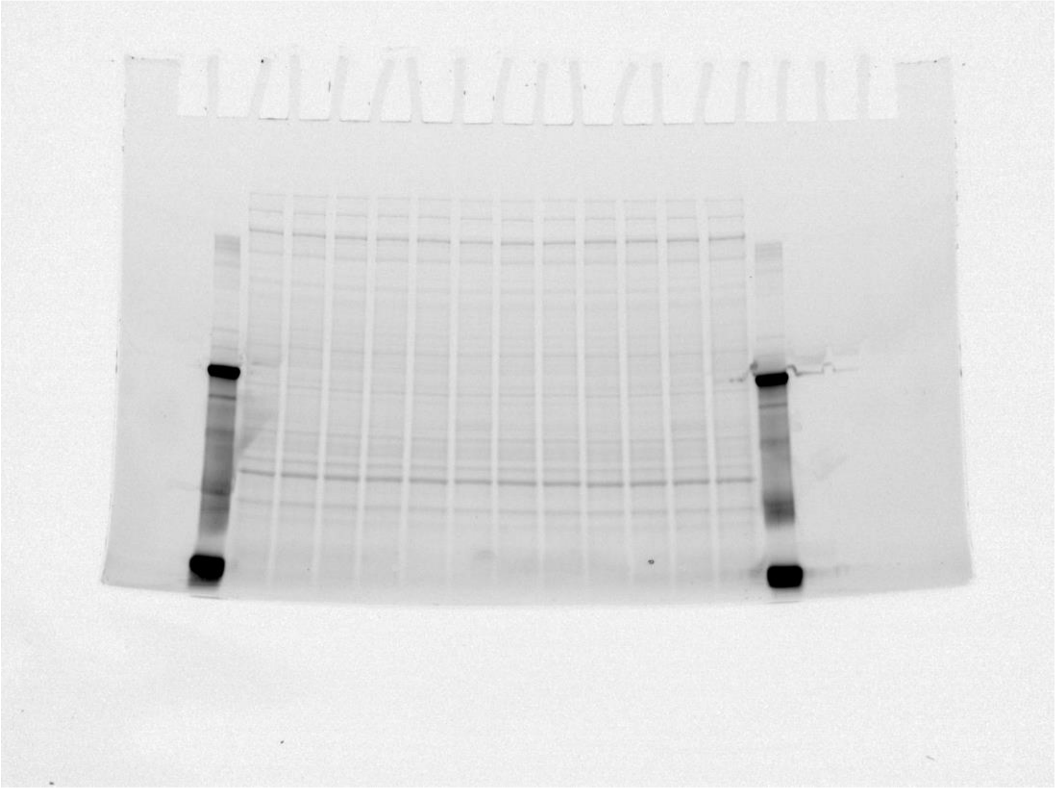

HMVEC-L

Replicate 3

HIF-3α2

0 Hypoxia (hours) 48

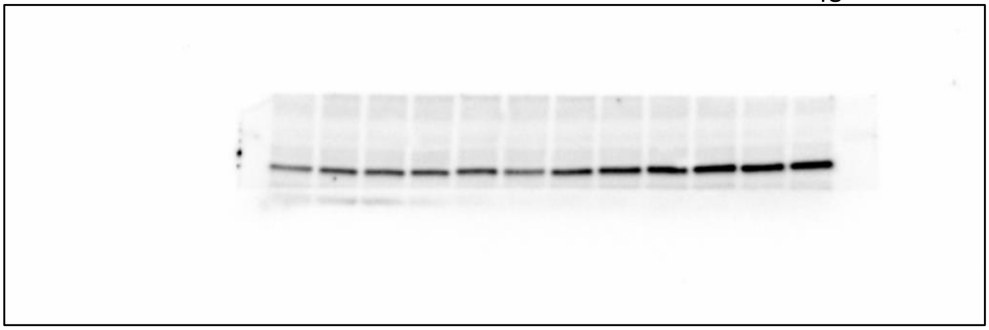

Actin β

0 Hypoxia (hours) 48

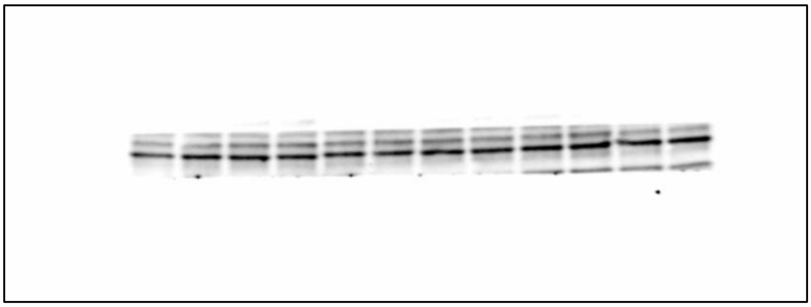

Total Protein

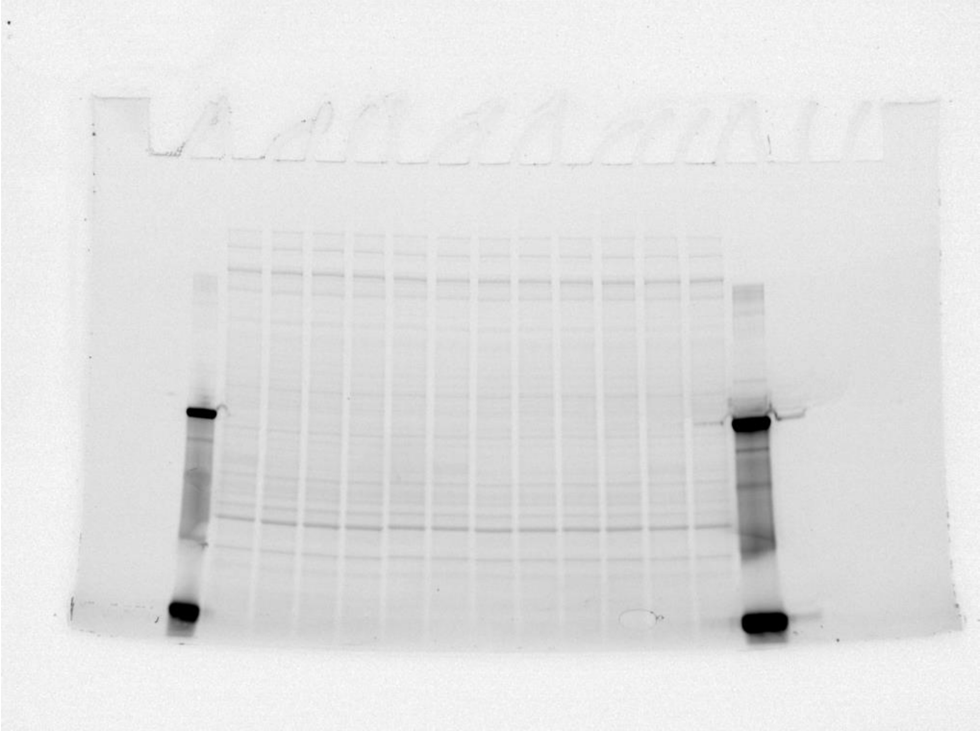

Supplement: Supplementary data PDF [file EXCLI-21-454-s-001.pdf]
